# *In Vitro* Epigenetic Reprogramming of Human Cardiac Mesenchymal Stromal Cells into Functionally Competent Cardiovascular Precursors

**DOI:** 10.1371/journal.pone.0051694

**Published:** 2012-12-17

**Authors:** Matteo Vecellio, Viviana Meraviglia, Simona Nanni, Andrea Barbuti, Angela Scavone, Dario DiFrancesco, Antonella Farsetti, Giulio Pompilio, Gualtiero I. Colombo, Maurizio C. Capogrossi, Carlo Gaetano, Alessandra Rossini

**Affiliations:** 1 Dipartimento di Scienze Cliniche e di Comunità, Università di Milano, Milano, Italy; 2 Laboratorio di Biologia Vascolare e Medicina Rigenerativa, Centro Cardiologico Monzino, IRCCS, Milano, Italy; 3 Istituto di Patologia Medica, Università Cattolica del Sacro Cuore, Roma, Italy; 4 Dipartimento di Bioscienze, The PaceLab, Università di Milano, Milano, Italy; 5 Istituto Nazionale Tumori Regina Elena, Roma, Italy; 6 Istituto di Neurobiologia e Medicina Molecolare, Consiglio Nazionale delle Ricerche (CNR), Roma, Italy; 7 Laboratorio di Immunologia e Genomica Funzionale, Centro Cardiologico Monzino, IRCCS, Milano, Italy; 8 Laboratorio di Patologia Vascolare, Istituto Dermopatico dell’Immacolata - IRCCS, Roma, Italy; 9 Division of Cardiovascular Epigenetics, Department of Cardiology, Goethe University, Frankfurt am Main, Germany; Tokai University, Japan

## Abstract

Adult human cardiac mesenchymal-like stromal cells (CStC) represent a relatively accessible cell type useful for therapy. In this light, their conversion into cardiovascular precursors represents a potential successful strategy for cardiac repair. The aim of the present work was to reprogram CStC into functionally competent cardiovascular precursors using epigenetically active small molecules. CStC were exposed to low serum (5% FBS) in the presence of 5 µM all-trans Retinoic Acid (ATRA), 5 µM Phenyl Butyrate (PB), and 200 µM diethylenetriamine/nitric oxide (DETA/NO), to create a novel epigenetically active cocktail (EpiC). Upon treatment the expression of markers typical of cardiac resident stem cells such as c-Kit and MDR-1 were up-regulated, together with the expression of a number of cardiovascular-associated genes including KDR, GATA6, Nkx2.5, GATA4, HCN4, NaV1.5, and α-MHC. In addition, profiling analysis revealed that a significant number of microRNA involved in cardiomyocyte biology and cell differentiation/proliferation, including miR 133a, 210 and 34a, were up-regulated. Remarkably, almost 45% of EpiC-treated cells exhibited a TTX-sensitive sodium current and, to a lower extent in a few cells, also the pacemaker I_f_ current. Mechanistically, the exposure to EpiC treatment introduced global histone modifications, characterized by increased levels of H3K4Me3 and H4K16Ac, as well as reduced H4K20Me3 and H3s10P, a pattern compatible with reduced proliferation and chromatin relaxation. Consistently, ChIP experiments performed with H3K4me3 or H3s10P histone modifications revealed the presence of a specific EpiC-dependent pattern in c-Kit, MDR-1, and Nkx2.5 promoter regions, possibly contributing to their modified expression. Taken together, these data indicate that CStC may be epigenetically reprogrammed to acquire molecular and biological properties associated with competent cardiovascular precursors.

## Introduction

Cellular cardiomyoplasty is a promising therapy to reconstitute injured hearts. Cell based interventions aimed at structurally regenerating the heart imply that transplanted cells graft in the host tissue and adopt the phenotype of resident cardiomyocytes, endothelial cells and smooth muscle cells. In this light, cells possessing pluripotency, such as embryonic stem (ES) cells and the so-called induced-pluripotent stem cells (iPS) [1] may be considered a good candidate. Yet, although several attempts have been made to simplify iPS cell generation methods avoiding the undesired effect of neoplastic transformation [2], their use still raises safety concerns. Consequently, much effort has been put into promoting cardiovascular differentiation of adult cells. Intriguingly, a recent work has shown the possibility of directly converting neonatal or embryonic mouse fibroblasts into cardiomyocytes by a transcription-factor based reprogramming strategy [3]. In spite of its potential practical relevance, the efficiency of this procedure is low and genetic manipulation of target cells is still required. In this context, chemical strategies based on the use of small active molecules represent an easier, more effective and safer alternative to genetic methods [4]. Further, achieving terminal differentiation of adult somatic or stem cells into cardiomyocytes may not be the right therapeutic approach, as multiple cell types (i.e. cardiomyocytes, vascular cells, and fibroblasts) should be generated to rebuild damaged heart tissue. In this perspective, reprogramming of adult cardiac cells into progenitors, which are less de-differentiated than iPS cells and exhibit lineage commitment restricted to the cell types of interest, may represent a successful strategy.

In addition to adult cardiac stem cells, a different category of heart cells, namely cardiac mesenchymal stromal cells (CStC) deriving from the cardiac parenchyma, have been recently isolated and characterized by our group [5]. CStC are c-Kit negative, reveal positivity for both pericytes (i.e. CD146) and fibroblast markers (i.e. vimentin and human fibroblast surface antigen) and share similarities with syngeneic bone marrow mesenchymal stromal cells (BMStC), showing comparable morphology and expression of mesenchymal-associated antigens (i.e. CD105, CD73, CD29, and CD44). Despite their similarities, significant differences between CStC and BMStC emerged. In fact, CStC may be identified by the expression of a specific microRNA signature and exhibit a residual plasticity toward the cardiovascular lineage, possessing the ability to contribute new adult-like cardiomyocytes after heart ischemia with higher efficiency than BMStC [5]. Of note, CStC are easily obtained from small biopsyspecimens and may be efficiently grown *in vitro*.

A large number of evidences indicate that biological response modifiers, including epigenetically active small molecules, such as histone deacetylase inhibitors (HDACi), may facilitate the redirection of adult cellular functions toward stemness [4]. So far, however, no reportsdescribed the *in vitro* enhancement of adult cardiac precursors via a defined cocktail of drugs.

In this report, we describe the properties of CStC chemically converted into functional cardiovascular precursors by means of nutrients deprivation in the presence of retinoids, phenyl butyrate and nitric oxide drugs. Remarkably, these drugs have been used in the past to stimulate *in vitro* cardiomyocyte production in different experimental contexts [6], [7], [8], but they were never combined together on cells isolated from human adult heart. These compounds have different mechanisms of action including nuclear receptor activation (retinoic acid) [9] and inhibition of histone deacetylases (phenyl butyrate). Further, nitric oxide donors such as DETA/NO, have been shown to play a role in the prevention of apoptosis, microRNA up-regulation [10] and cardiac commitment [11]. Therefore it is possible that these drugs may synergize in determining the generation of functionally competent cardiovascular precursor-like cells.

## Materials and Methods

### Ethics Statement

Cardiac stromal cells were obtained from right auricle samples of donor patients undergoing valve substitution or by-pass surgery after signed informed consent and approval by the Centro Cardiologico Monzino (Milano, Italy) Ethical Committee. Investigations were conducted according to the principles expressed in the Declaration of Helsinki. Data were analyzed anonymously.

### CStC Isolation and Culture

CStC were isolated from right auricles and cultured in growth medium (GM) as previously described [5]. CStC at passages 4–8 were incubated for 7 days with an Epigenetic cocktail (EpiC), composed by Iscove’s Modified Dulbecco’s Medium (IMDM), 5% Foetal Bovine Serum (FBS), 10.000 U/ml Penicillin/Streptomicin, 10 mg/ml L-Glutamine, 5 µM All trans Retinoic Acid (ATRA), 5 µM Phenyl Butyrate (PB), and 200 µM diethylenetriamine/nitric oxide (DETA/NO), all purchased from Sigma Aldrich. The EpiC medium was changed every 48 hrs.

### Western Blot Analysis

CStC in control and EpiC media were lysed with Laemmli buffer in presence of protease and phosphatase inhibitors (Roche Diagnostic). Proteins were resolved by SDS–PAGE, transferred onto nitrocellulose membranes (Bio-Rad Laboratories), and incubated overnight at 4°C with primary antibodies listed in Supplementary Table S1. Subsequently, the blots were incubated with the appropriate anti-rabbit, anti-mouse, or anti-goat horseradish peroxidase-conjugated secondary antibody (Amersham-GE Healthcare). ECL or, when appropriate, ECL plus (Amersham-GE Healthcare) were used for chemiluminescence detection. Each filter was also probed with anti-β-actin, anti-β-tubulin, anti-H3, or anti-H4, to verify equal protein loading. Densitometric analyses were performed by NIH ImageJ software, version 1.4.3.67.

### Real-time Reverse Transcription–polymerase Chain Reaction Analysis

Total RNA was extracted from cells using the TRIzol reagent and 500 ng of RNA were reverse-transcribed using Superscript III reverse transcriptase (Invitrogen). cDNA was amplified by SYBR-GREEN quantitative PCR in an iQ5 Cycler (Bio-Rad Laboratories). Primer sequences are reported in Supplementary Table S2. Relative expression was estimated using the DeltaCt (ΔCt) method. ΔΔCt were calculated using average ΔCt for each gene expression in GM. Fold changes in gene expression were estimated as 2^(−ΔΔCt)^. ΔCt = 25 was arbitrarily assigned to non-expressed genes.

### microRNA Profiling Analysis

Total RNA was extracted from cells using TRIzol reagent (Invitrogen), according to the manufacturer’s instructions. The concentration and purity of RNA were determined using a NanoDrop 1000 spectrophotometer (Thermo Scientific), and only highly pure preparations (ratio of 260/280>1.8 and 260/230>1.8) were used. The integrity of total RNA was assessed using an Experion electrophoresis system and the RNA high sense Analysis Kit (Bio-Rad Laboratories) and only highly quality RNA (RQI >9.5/10) was subjected to subsequent analysis. Comparative microRNA expression profiling was carried out using TaqMan Low Density Arrays Human MicroRNA A+B Cards Set v3.0 (Applied Biosystems). All procedures were performed according to the manufacturer’s instructions, on a 7900HT Real-Time PCR System (Applied Biosystems).

Quality control and low level analysis of TaqMan Arrays were performed using the software ABI Prism SDS v2.4. Optimal baseline and Ct (threshold cycle) were determined automatically by the software algorithm. All Ct values reported as greater than 35 or as not detected were changed to 35 and considered negative calls. Raw expression intensities of target microRNAs were normalized for differences in the amount of total RNA added to each reaction using the mean expression value of all expressed microRNAs in a given sample, following the method described by Mestdagh and co-workers [12]. Relative quantitation of microRNA expression was performed using the comparative Ct method (ΔCt). ΔCt values were defined as the difference between the Ct of any microRNA in the calibrator sample (the sample with the highest expression, i.e. lowest Ct value) and the Ct of the same microRNA in experimental sample. The Ct values were transformed to raw quantities using the formula η^ΔCt^, where amplification efficiency (η) was set arbitrarily to 2 (100%). The normalized relative expression quantity of each microRNA was calculated by dividing its raw quantity by the normalization factor. MicroRNAs that had missing values in greater than 50% of the samples (i.e. that were not present at least in all cells on one treatment) were deemed as uninformative and removed from the dataset.

### Chromatin Immunoprecipitation (ChIP) Assay

ChIP assays were performed and DNA fragments analyzed by quantitative real-time PCR (qPCR) as previously described [13]. Briefly, standard curves were generated by serially diluting the input DNA (5-log dilutions in triplicate) and qPCR was done on an ABI Prism 7500 PCR instrument (Applied Biosystems), using a SYBR Master mix (Applied Biosystems) and evaluating the dissociation curves. The qPCR analyses were performed in duplicate and the data obtained were normalized to the corresponding DNA input. Data are represented as relative enrichment of specific histone modifications in EpiC-treated cells compared to growth medium (GM). Primers for human promoters were (position from transcriptional starting site, TSS): c-Kit fw: GAGCAGAAACAATTAGCGAAACC (−560 bp); c-Kit rev: GGAAATTGAGCCCCGACATT (−468 bp); Nkx2.5 fw: TGACTCTGCATGCCTCTGGTA (−198 bp); Nkx2.5 rev: TGCAGCCTGCGTTTGCT (−138 bp); MDR-1 fw: TTCCTCCACCCAAACTTATCCTT (-93 bp); MDR-1 rev: CCCAGTACCAGAGGAGGAGCTA (−2 bp); hGNL3 fw: GAGTTTGTGTCGAACCGTCAAG (−563 bp); hGNL3 rev: TCCCTCAGTCCCCAATACCA (−457 bp).

### Electrophysiology

Patch-clamp analysis was performed on CStC perfused with a normal Tyrode solution containing (mM): 140 NaCl, 5.4 KCl, 1.8 CaCl_2_, 5.5 D-glucose, 5 Hepes-NaOH; pH 7.4. Patch pipettes were filled with a solution containing (mM): 130 K-Aspartate, 10 NaCl, 5 EGTA-KOH, 2 CaCl_2_, 2 MgCl_2_, 2 ATP (Na-salt), 5 creatine phosphate, 0.1 GTP (Na-salt), 10 Hepes-KOH; pH 7.2 and had resistances of 2 to 4 MOhm. Experiments were carried out at room temperature.

The fast Na+ current (I_Na_) was activated by 50 ms steps to the range −80/+30 mV from a holding potential of −90 mV. Peak I-V relations were constructed by plotting the normalized peak current against test voltages. The time-independent inwardly-rectifying K+ current (I_K1_) was investigated by applying 4 s voltage-ramps from −100 to 25 mV in Tyrode solution and after addition of Ba^2+^ (2 mM BaCl_2_), a known blocker of I_K1_. To record the I_f_ current, 1 mM BaCl_2_ and 2 mM MnCl_2_ were added to normal Tyrode in order to block contaminating currents. I_f_ was activated by a standard activation protocol [14]. Hyperpolarizing test steps to the range −35/−125 mV were applied from a holding potential of −30 mV, followed by a fully activating step at −125 mV. Each test step was long enough to reach steady-state current activation. Normalized tail currents measured at −125 mV were used to plot activation curves, which were fitted to the Boltzmann distribution function: y = (1/(1+exp((V-V_1/2_)/s), where V is voltage, y fractional activation, V_1/2_ the half-activation voltage, and s the inverse slope factor. Measured values are reported as mean ± SEM.

### Statistics

Statistical analysis of real-time PCR, Western Blot, and HDAC Class I activity data was performed using Student’s *t*-test. A P<0.05 was considered significant.

Statistical analysis of the TaqMan Arrays was performed using the MultiExperiment Viewer (MeV) software v4.8.1 [15]. microRNAs with a fold change between the two treatment groups less than ±2 were filtered out. Normalized expression values were log_2_ transformed and differentially expressed microRNAs were identified using a paired *t*-test computing p-values based on all available permutations with a confidence level of 95% and limiting the false discovery rate (FDR) proportion to <0.15. Differences in microRNA expression were considered statistically significant if their P-value was <0.05. Unsupervised hierarchical cluster analysis was performed to assess whether differential profile discriminates the EpiC from the control treated cells. The similarity of microRNA expression among arrays and probes was assessed by calculating the Pearson’s correlation (uncentered) coefficient. Normalized log_2_ transformed expression values were mean centered and clustered by correlation average linkage, using leaf order optimization.

### Supplementary Methods

An additional Methods section is available as Supporting Information Document S1.

## Results

### Epigenetic Cocktail (EpiC) Design

Human cardiac stromal cells (CStC) cultured in standard medium for mesenchymal cells (GM) are positive for the mesenchymal markers CD105, CD29 and CD73, but negative for adult cardiac stem cell markers Sca-1, c-Kit and VEGFR2 (Figure S1A).

**Figure 1:**
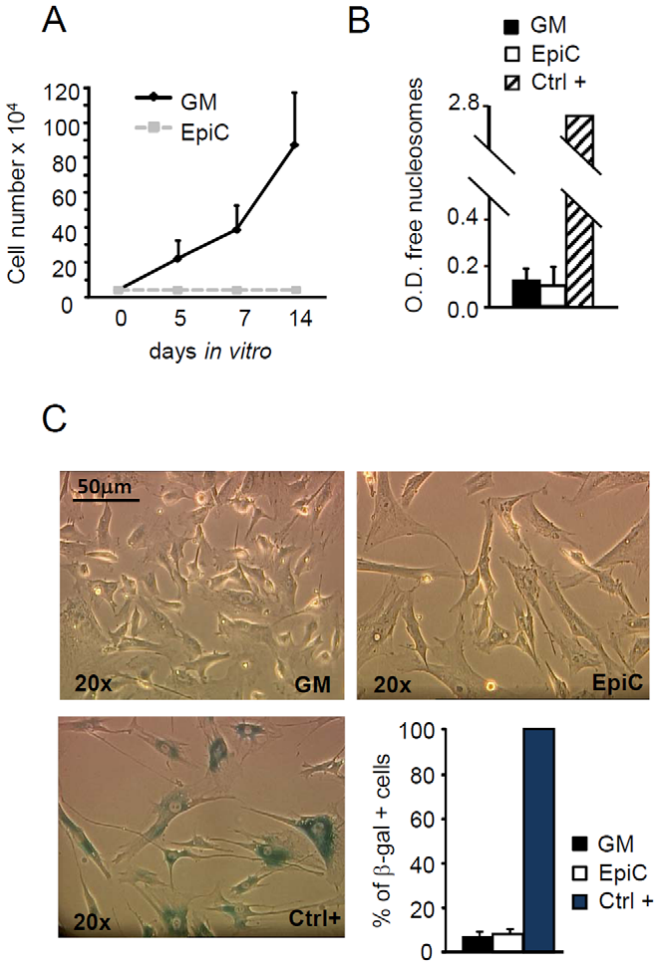
Effect of EpiC treatment on CStC growth, viability and senescence. (A) Growth curve of CStC cultured in GM or EpiC for 5, 7, and 14 days (n = 7). (B) Quantification of free nucleosomes, used as markers of apoptosis, in CStC exposed to GM or EpiC for7 days. Ctrl+ = positive control supplied by the manufacturer. (C) Staining for senescence-related acidic β-galactosidase (β-gal ) performed on CStC grown either in GM or in EpiC medium. Positivity for β-gal is indicated by the presence of a dark grey stain within the cytoplasm. Ctrl+ = primary bone marrow mesenchymal stromal cells at passage 10 (replicative senescence). Bar Graph shows average percentage of β-gal positive cells (n = 4). Original magnification: 20×.

The level of nutrients (i.e. foetal serum) and the presence of selected drugs can modify cell phenotype and fate inducing functional reprogramming [4]. In light of this, after expansion in a medium routinely used for mesenchymal cell culture (growth medium, GM), CStC were exposed for 7 days to a medium with reduced level of foetal bovine serum (5% FBS) either in the presence or in the absence of 5 µM all-trans retinoic acid (ATRA), 5 µM phenyl butyrate (PB) and 200 µM diethylenetriamine/nitric oxide (DETA/NO), alone or in combination. In all these conditions, the expression of markers associated with resident cardiac stem cells (c-Kit, VEGFR2, and MDR-1) [16], [17] has been evaluated. Our findings indicated that, although serum deprivation alone or a single drug exhibited the ability to up-regulate the expression of one or more markers, only the complete formulation, defining a novel “epigenetic cocktail” (EpiC), induced the coincident expression of c-Kit, VEGFR2, and MDR-1 in CStC (Figure S1B). Notably, EpiC treatment, while stopping cell proliferation, did neither induce apoptosis or senescence (Figure 1), nor stimulate CStC to differentiate into other mesodermal cells such as adipocytes or osteoblasts (Figure S1C), nor modulated the expression of Sca-1 and typical mesenchymal markers such as CD105 (not shown).

### Effects of EpiC Treatment on Stem and Cardiovascular Precursor Markers

**Figure 2:**
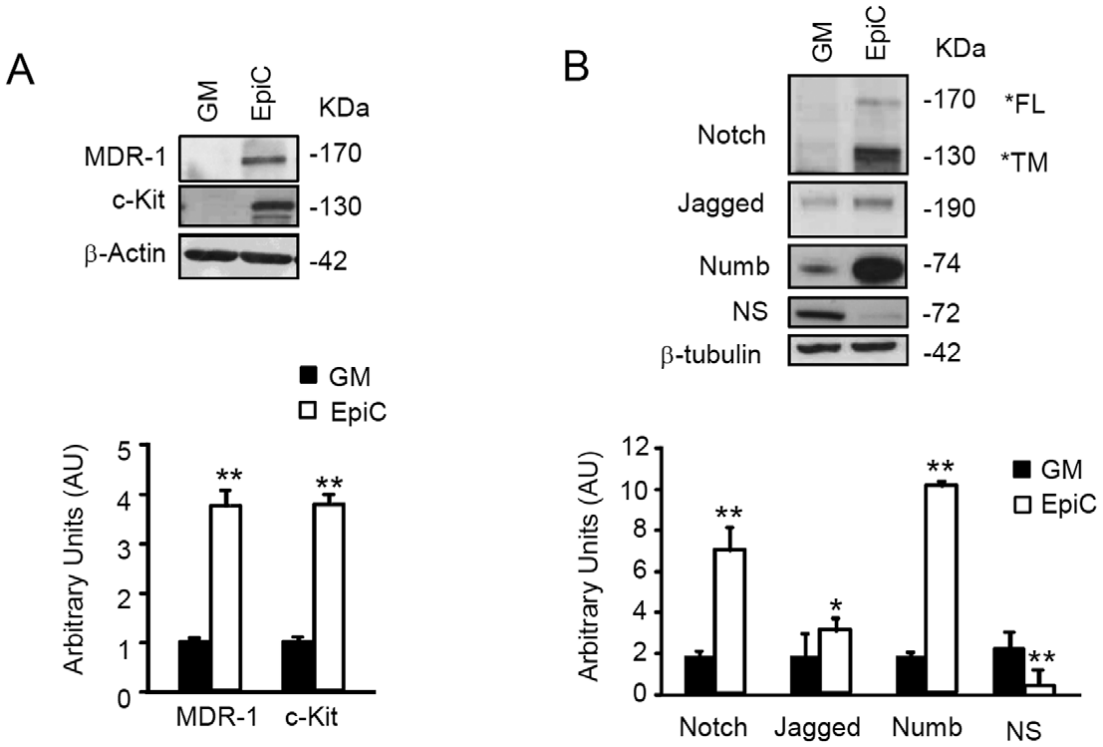
Effect of EpiC treatment on stem cell marker expression. Western blotting analysis of (A) adult cardiac stem cell marker c-Kit and MDR-1 (n = 4) and (B) Notch, Jagged-1, Numb and nucleostemin (NS) in CStC, grown either in GM or in EpiC (n = 6). Densitometry is shown in the bar graphs at the bottom of each panel (**P≤0.01, *P≤0.05 vs GM). FL = full length protein; TM = Transmembrane domain.

**Figure 3:**
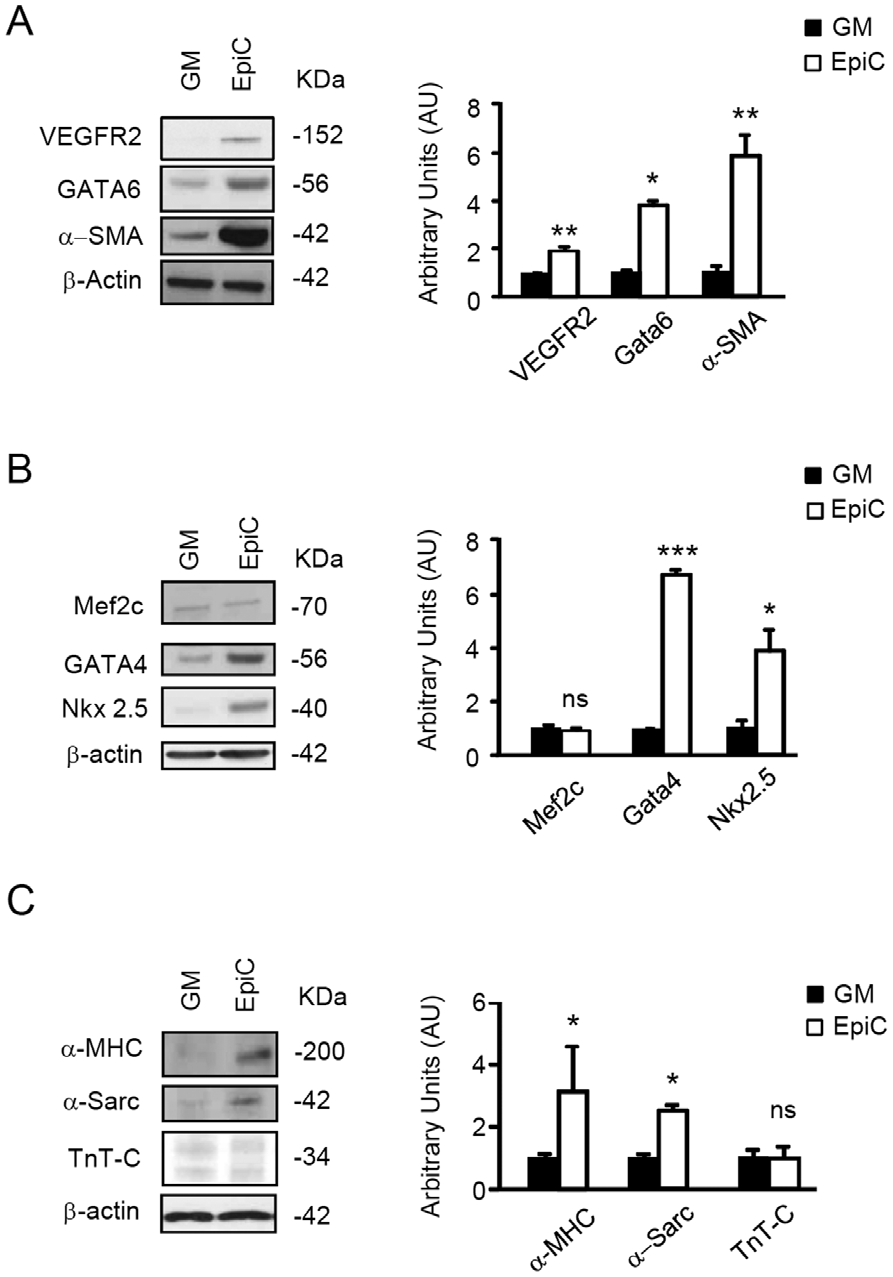
Effect of EpiC treatment of cardiovascular marker expression. Immunoblots showing expression analyses of (A) vascular markers: VEGFR-2, GATA6, and α-smooth muscle actin (α-SMA); (B) early cardiomyogenic markers: Mef2c, GATA4, and Nkx2.5; (C) late cardiomyogenic markers α-MHC, α-Sarc, and Tn-TC in EpiC-treated CStC compared to cells in GM. Bar graphs represent average results, normalized to β-actin, of western blot densitometric analyses (*P≤0.05, **P<0.01, and ***P≤0.005, vs GM). The data represent the mean ± SD of 4 independent experiments performed in duplicate.

EpiC treatment of CStC strongly up-regulated markers associated to cardiac resident adult stem cell such as c-Kit and MDR-1 (Figure 2A and Figure S2A). In this condition, we were able to demonstrate that the MDR-1 transporter was functionally active as indicated by the rhodamine extrusion assay (Figure S2B). Other proliferation and differentiation markers including Notch, Jagged-1 and Numb [18], [19] were also increased (Figure 2B), while the expression of the pluripotency factors Oct4 and Nanog remained negative (not shown). In this condition, EpiC-treated cells were growth arrested (Figure 1A) and nucleostemin (NS), a nucleolar protein present in proliferating stem cells [20], was down-regulated (Figure 2B). Of note, untreated CStC expressed detectable level of GATA6, α-smooth muscle actin and GATA4, whose expression is associated with processes ongoing during vascular and cardiac commitment [21], [22], [23]. Interestingly, EpiC treatment increased the expression of markers for both vascular (VEGFR2, GATA6, and α-smooth muscle actin) and cardiomyogenic (GATA4 and Nkx2.5) precursors, while leaving Mef2C expression unaltered (Figure 3A and 3B). However, in spite of the evidence that more mature cardiomyogenic markers could be detected in EpiC-treated CStC, such as α-sarcomeric actin (α -Sarc) and α-myosin heavy chain (α -MHC), neither sarcomere striation nor increased cardiac troponin (TnT-C) expression were observed (Figure 3C).

### Effects of EpiC Treatment on CStC MicroRNA Expression Profile

**Figure 4:**
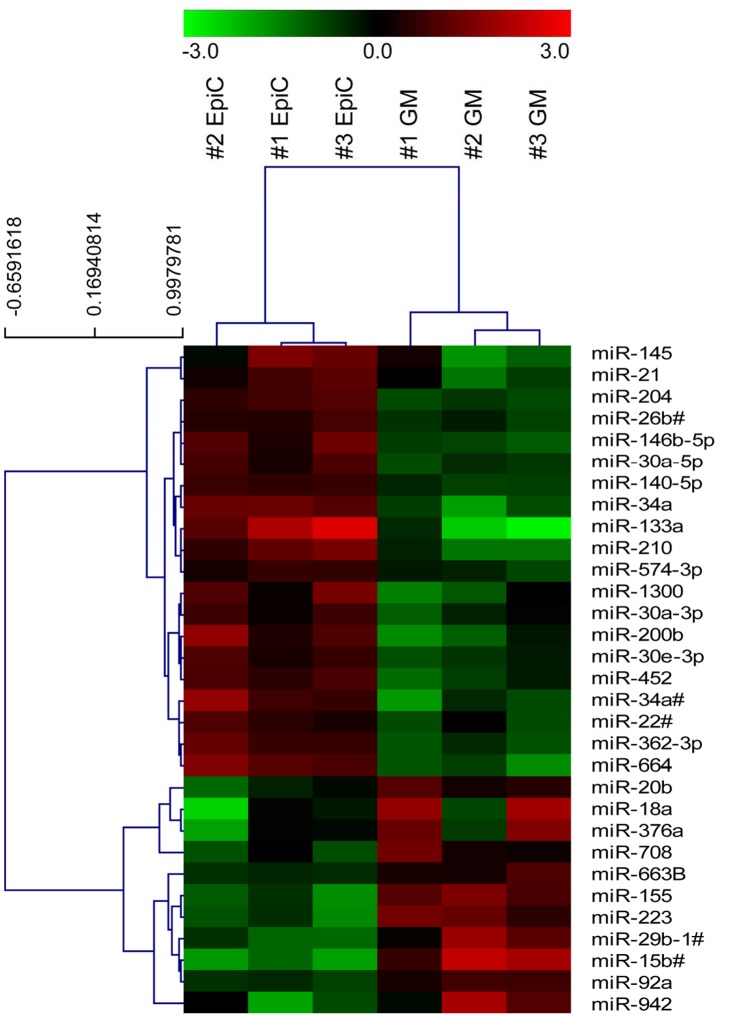
Hierarchical clustering of differentially expressed microRNAs in GM and EpiC-treated CStC. Unsupervised cluster analysis was performed using the 31 microRNAs that showed a significant modulation induced by the EpiC treatment. The dendrogram above shows that the differential expression profile completely discriminates the two treatment groups. The dendrogram on the left shows two distinct clusters of microRNAs up- and down-regulated by EpiC treatment in CStC (respectively at the top and the bottom of the heatmap). The mean centered value of normalized log_2_ transformed relative expression level of each microRNA is represented with a green, black, and red color scale (green indicates below mean; black, equal to mean; and red, above mean).

microRNAs (miR) are 22–23 nucleotide-long, single-stranded ribonucleic molecules usually involved in transcriptional repression and gene silencing [24]. As miR are known to have important roles in cell reprogramming [25] and cell differentiation [26], their expression profile was evaluated in CStC after 7 days of exposure to EpiC. Two hundred and sixty-one microRNAs passed the quality assurance and filtering criteria: their normalized relative expression levels and fold-differences between EpiC and GM-treated cells, along with the raw P-values, are reported in Supplementary Table S3. Results of the differential analysis (Table 1) showed that 31 microRNAs were significantly modulated by EpiC treatment. In particular, 20 microRNAs were up-regulated >2-fold (among which miR-133a, miR-34a, and miR-210) and 10 were down-regulated (such as miR-155). Unsupervised hierarchical cluster analysis was performed using the whole dataset of 261 microRNAs, revealing that the global expression profile well discriminates between the two groups of treatment (Figure S3). Likewise, unsupervised cluster analysis of the differentially expressed microRNAs correctly discriminated between EpiC and control treated CStC, sorting them into two independent groups (Figure 4). In addition, it showed two clusters of microRNAs with highly correlated expression, the first increased and the latter decreased in EpiC-treated cells, which may be deemed as a specific signature of co-regulated microRNAs.

**Table 1:** Differentially expressed microRNAs.

microRNA	GM	EpiC	FC	P-value	FDR
**miR-133a**	0.07±0.07	0.86±0.43	11.88	0.0488	0.13
**miR-34a**	0.26±0.10	1.23±0.13	4.78	0.0266	0.12
**miR-664**	0.30±0.09	1.44±0.34	4.75	0.0035	0.06
**miR-34a#**	0.31±0.13	1.33±0.61	4.27	0.0209	0.10
**miR-210**	0.25±0.10	1.04±0.29	4.19	0.0266	0.11
**miR-200b**	0.33±0.16	1.34±0.63	4.06	0.0492	0.13
**miR-146b-5p**	0.29±0.04	1.00±0.31	3.42	0.0442	0.14
**miR-145**	0.31±0.23	0.95±0.47	3.09	0.0346	0.13
**miR-362-3p**	0.48±0.10	1.50±0.35	3.09	0.0002	0.02
**miR-1300**	0.31±0.18	0.93±0.38	3.02	0.0126	0.09
**miR-204**	0.38±0.03	1.14±0.16	2.99	0.0124	0.09
**miR-452**	0.42±0.13	1.20±0.17	2.87	0.0113	0.10
**miR-30a-5p**	0.43±0.05	1.13±0.23	2.63	0.0056	0.08
**miR-21**	0.40±0.19	1.02±0.29	2.52	0.0478	0.13
**miR-140-5p**	0.52±0.07	1.28±0.05	2.46	0.0114	0.09
**miR-30e-3p**	0.55±0.12	1.31±0.27	2.37	0.0181	0.10
**miR-26b#**	0.49±0.07	1.09±0.18	2.22	0.0423	0.14
**miR-22#**	0.64±0.25	1.40±0.31	2.19	0.0162	0.09
**miR-30a-3p**	0.56±0.19	1.14±0.25	2.03	0.0140	0.09
**miR-574-3p**	0.60±0.11	1.21±0.16	2.02	0.0445	0.13
**miR-663B**	1.32±0.34	0.63±0.02	−2.08	0.0397	0.14
**miR-20b**	1.28±0.34	0.58±0.20	−2.21	0.0455	0.13
**miR-708**	1.07±0.51	0.46±0.19	−2.34	0.0020	0.04
**miR-92a**	1.58±0.27	0.68±0.07	−2.34	0.0303	0.12
**miR-376a**	1.15±0.70	0.43±0.23	−2.71	0.0093	0.09
**miR-942**	1.38±0.92	0.36±0.22	−3.82	0.0009	0.04
**miR-18a**	1.16±0.79	0.30±0.19	−3.91	0.0072	0.08
**miR-223**	1.35±0.38	0.32±0.12	−4.17	0.0017	0.05
**miR-29b-1#**	1.51±0.82	0.35±0.09	−4.38	0.0282	0.11
**miR-155**	1.83±0.43	0.40±0.15	−4.54	0.0206	0.10
**miR-15b#**	1.53±0.75	0.15±0.04	−10.42	0.0429	0.14

microRNA normalized relative expression levels in GM and EpiC-treated cells are expressed as mean ± SD. FC = fold change. FDR = false discovery rate.

### EpiC Treatment Changes CStC Electrophysiological Properties

**Figure 5:**
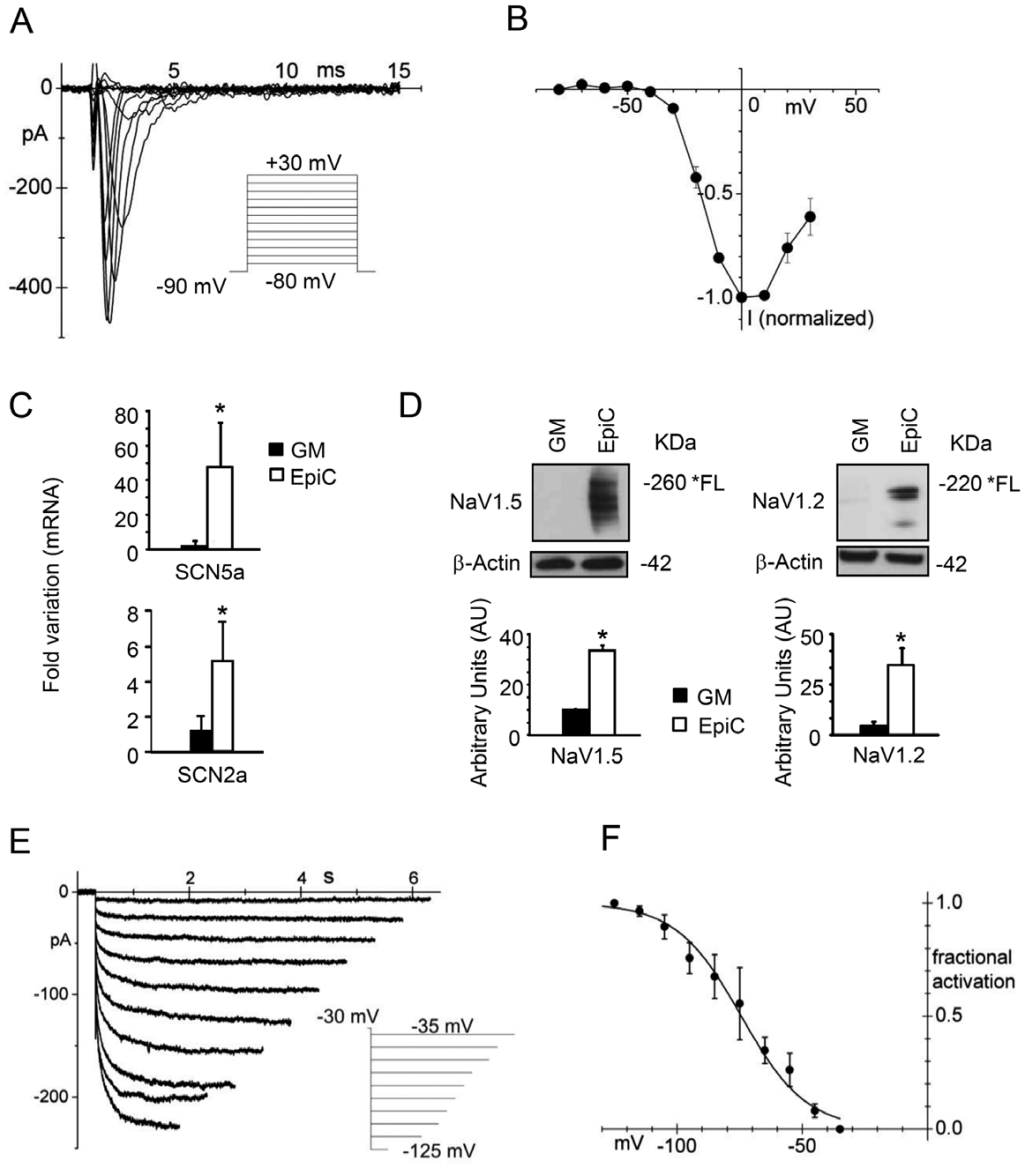
EpiC-treated CStCs express functionally competent ion channels. In panel (A) a family sodium current recorded from a representative EpiC-treated TTX (10 µM) low -sensitive CStC, following the application of a standard depolarizing protocol (see inset), is shown. (B) Mean current-voltage relation of normalized TTX-sensitive currents showing a threshold of activation around −40 mV and a peak around 0 mV. (C) SCN5A and SCN2A genes were up-regulated (n = 3, *P≤0.05) by real-time PCR analysis. (D) Western Blot evidences increase of the type V (NaV1.5) and type II (NaV1.2) voltage-gated sodium channel protein level in CStC cells exposed to EpiC (n = 5, *P≤0.05). FL = full length protein. (E) In few cells, membrane hyperpolarization in the range −35 to −125 mV (see inset) revealed an inward current with the kinetic features of the native pacemaker I_f_ current. (F) Plot of the mean activation curve obtained from the analysis of the I_f_ currents recorded in a subset of EpiC-treated CStC.

The exposure to EpiC had a profound impact on CStC function, determining the appearance of electrophysiological features typical of cells committed towards the cardiomyocyte lineage. Specifically, a significant fraction (44.8%, 13 out of 29 cells) of EpiC-treated cells exhibited a fast-activating inward sodium current (Figure 5A), which activated at voltages more positive than −40 mV, peaked around 0 mV, and was completely blocked by TTX (10 µM, Figure 5B). At a lower concentration (50 nM) TTX had variable effects on sodium current. In fact, in a group of cells, sodium current was not blocked at all (n = 3) while in another group the block ranged from 38 to 94% (mean value: 76.3±9.2%, n = 3, data not shown). These results suggest that EpiC-treated cells may present both TTX-sensitive and TTX-resistant sodium currents. Accordingly, expression analyses showed that, in the presence of EpiC, the type V (NaV1.5) and type II (NaV1.2) voltage-gated sodium channels (known for having different TTX sensitivity and encoded by the SCN5A and the SCN2A genes, respectively) were significantly up-regulated, both at the mRNA (Figure 5C) and at the protein level (Figure 5D). A pacemaker I_f_ current (Figure 5 E and F) has also been recorded in 5 out of 33 EpiC-treated cells (15.1%), with kinetic properties compatible with those of immature cardiomyocytes and of native pacemaker cells (V_1/2_: −74.4±5.7 mV, n = 3; Figure 5E and F) [27]. Accordingly, EpiC increased the expression of the pacemaker channel subunit HCN4 (Figure S4). Notably, CStC maturation towards the cardiomyocyte lineage was far from being complete, as demonstrated by the absence of the intracellular Ca2+ handling proteins NCX1 and RyR2 (not shown) and the depolarized resting potential characterizing CStC exposed to EpiC (−12.7±2.7 mV, n = 7). This last observation is in accordance with the negligible expression of the inward rectifying I_K1_ current (0.43±0.04 pA/pF at −100 mV, n = 5; not shown), physiologically important in setting the resting potential of working cardiomyocytes.

### EpiC Treatment Modulated CStC Epigenetic Landscape

**Figure 6:**
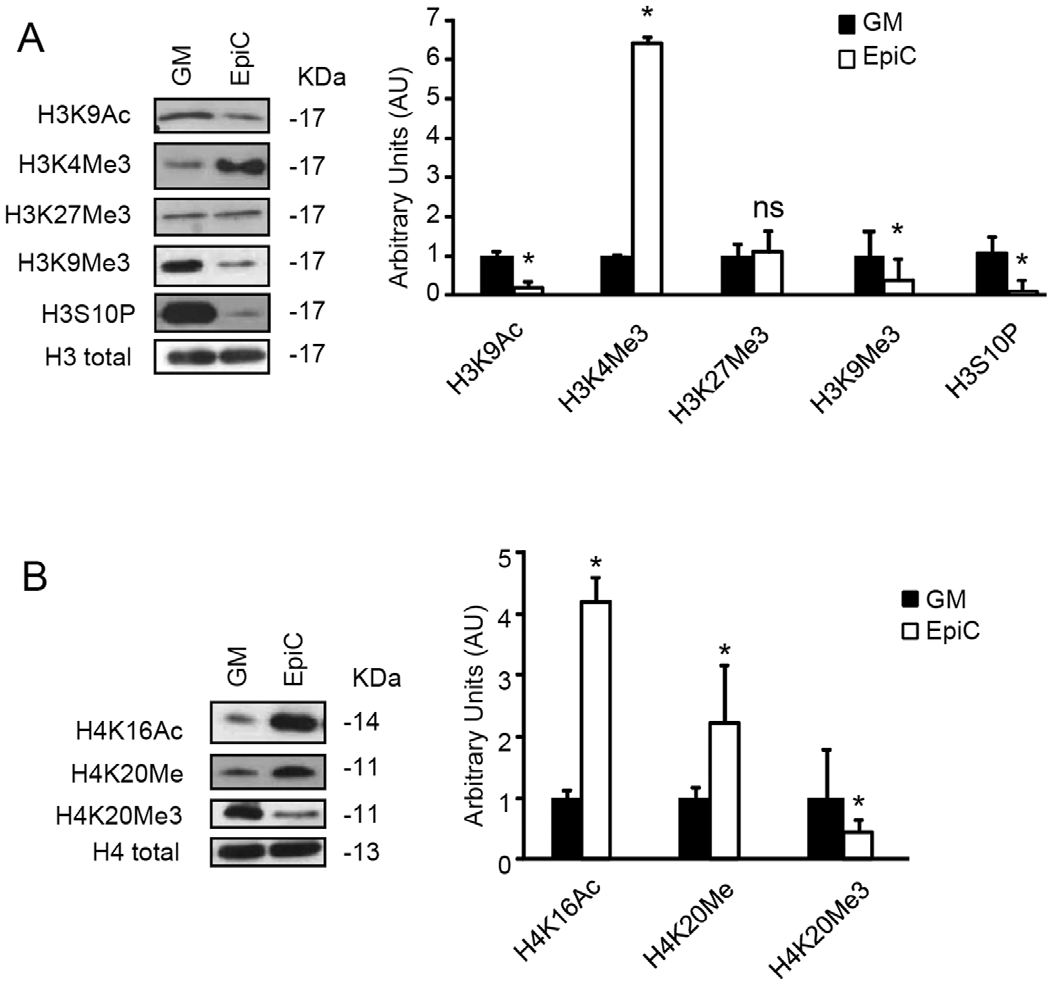
EpiC treatment modifies CStC epigenetic landscape. Western Blot analysis showing histone modification changes in EpiC-treated CStC compared to GM. (A) H3 modifications: H3K9Ac, H3K4Me3, H3K27Me3, H3K9Me3, and H3S10P. (B) H4 modifications: H4K16Ac, H4K20Me, and H4K20Me3. The same filter was probed with anti-total histone H3 or H4, respectively, to control for equal nuclear protein loading. Band densitometric analyses are reported in the bar graphs on the right (n = 3, *P≤0.05).

As expected by the composition of the EpiC described above, EpiC-treated CStC revealed a significantly lower HDAC Class I activity compared to cells kept in control condition (Figure S5) and changes in a number of genome wide histone modifications could be detected. Specifically, in agreement with its anti-proliferative effect EpiC also induced a significant decrease in Histone H3 phosphorylation at Ser10 (H3S10P, Fig. 6A) [28]. Further, we observed a global decrease in histone H3 lysine 9 trimethylation (H3K9Me3) and H4 lysine 20 trimethylation (H4K20Me3), paralleled by an increase in histone H4K20 monomethylation (H4K20Me, Fig. 6A and B). These modifications are compatible with an open chromatin structure and have been reported in cells undergoing differentiation or cell cycle arrest [29]. On the other hand, an increased density of the permissive marks histone H3K4 trimethylation (H3K4Me3) [30] and histone H4K16 acetylation (H4K16Ac) [31] was observed (Fig. 6 A and B), suggesting the presence of cells with a high developmental potential [32]. Along this line of evidence, repressive markers [33] remained stable (H3K27Me3) or were significantly down-modulated (H3K9Me3 and H4K20Me3), suggesting that EpiC-treated cells underwent a site-selective chromatin remodelling process leading to regulation of gene transcription.

### EpiC Introduces Chromatin Changes at Specific-gene Promoters

**Figure 7:**
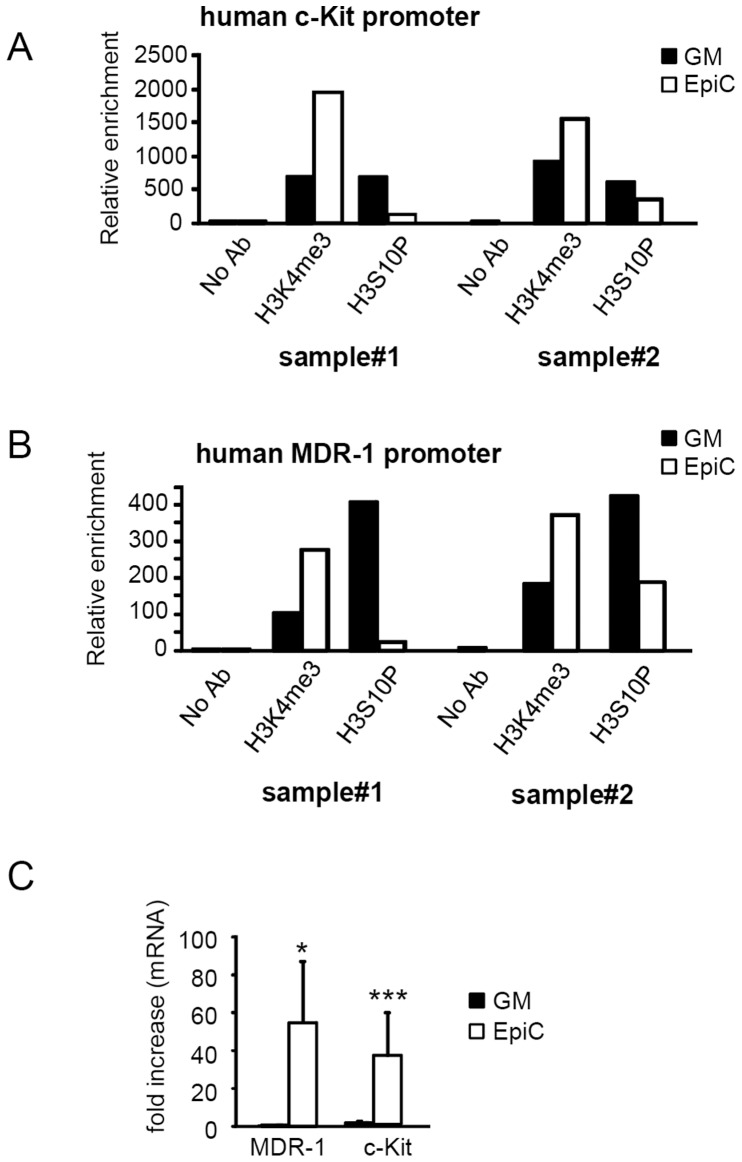
Effects of EpiC treatment on specific-gene promoters. ChIP experiments, performed on CStC isolated from 2 different patients, show that H3K4Me3 association to (A) c-Kit and (B) MDR-1 promoter is enriched after EpiC treatment, while H3S10P is decreased. Data are expressed as relative enrichment of specific histone modifications i in EpiC-treated cells compared to GM measured by real-time PCR amplification. (C) Real-time RT-PCR analysis of c-Kit and MDR-1 mRNA in EpiC-treated CStC (n = 3, *P≤0.05 and ****P≤0.005).

To validate the hypothesis that Epic may specifically regulate gene expression, a series of chromatin immunoprecipitation (ChIP) experiments were performed in CStC cultured in the presence or the absence of EpiC. Specifically, the highly divergent H3K4Me3 and H3S10P were used to immunoprecipitate chromatin, followed by real-time PCR to detect the relative modulation of these specific histone modifications in the promoter region of c-Kit, MDR-1, Nkx2.5 and nucleostemin. As shown in Figure 7A and B and in Figure S6A, H3K4Me3 association to c-Kit, MDR-1, and Nkx2.5 promoter was increased by EpiC treatment, suggesting that chromatin conformational modifications may account for the increased expression of these genes (Figure 7C). Accordingly, the presence of H3K4Me3 and H3S10P in the NKX-2.5 and nucleostemin (human GNL3 gene product) promoters was reduced, suggesting local structural changes as the basis of their down-regulation (Figure S6B).

## Discussion

Resident cardiac stem cells are specialized multipotent cells which, at very low rate, contribute to cardiomyocyte turn-over or cardiac post-ischemic regeneration. They are present in defined cardiac districts, identified as niches, in which these cells are kept quiescent until activation signals come [17]. Specific markers identify a cardiac stem cell, including the presence of c-Kit and the P-pump (MDR-1) gene products [16], [17]. Adult cardiac stem cells are neither easily grown *ex-vivo* nor effectively differentiated into mature cardiomyocytes. Further, the preparation of near-terminally differentiated cardiomyocytes may not be the best approach for applications aimed at cardiac regeneration where maximum plasticity is required to regenerate all the cell types forming damaged tissues.

Our group has recently isolated, characterized and efficiently amplified a human population of adult cardiac mesenchymal-like stromal cells (CStC) showing *in vitro* and *in vivo* cardiovascular plasticity [5]. Although CStC proneness to acquire cardiomyogenic markers was higher than that of syngeneic bone marrow cells, CStC efficiency to differentiate into adult cardiovascular cell types remained low, in spite of the expression of detectable mRNA encoding for early cardiovascular markers including c-Kit, GATA4 and GATA6 [5].

In the present work, CStC differentiation potential was improved by using a novel combination of small epigenetically active molecules, defined here as epigenetic cocktail, or “EpiC”. This cocktail was designed to modify the CStC chromatin landscape and, thus, to unmask and drive CStC plasticity possibly towards the cardiovascular lineage. In detail, EpiC was made of: (*i*) all-trans retinoic acid (ATRA), which has genome-wide regulation properties [34] and whose receptors are known to recruit p300 and CBP acetyl-transferases facilitating their action at the histone level of specific gene loci [35]. Relevant for this study, retinoic acid retains well known morphogenetic properties including a profound effect on heart development and regeneration [36]; (*ii*) phenyl butyrate (PB), a drug belonging to the family of histone deacetylase inhibitors, which are known to enhance mesoderm maturation [37]; (*iii*) diethylenetriamine/nitric oxide (DETA/NO), a nitric oxide donor associated to cell survival growth arrest in vascular cells [38] and increased mesendoderm differentiation in mouse ES cells [10]; (*iv*) a reduced serum content, known to induce spontaneous differentiation in a variety of cell types including C2C12 myoblasts [39] or cardiac mesoangioblasts [40]. Importantly, all drugs used in this study are approved for clinical use or, in case of DETA/NO, currently undergoing clinical trials.

The idea that stem cell fate can be modulated by specific chemicals dates back decades but, recently, an increasing number of studies are showing the potential role of small molecules to promote cardiogenesis in mouse ES cells including different BMP inhibitors such as dorsomorphin [41] and Wnt pathway modulators [42]. On the other hand, similar attempts on adult cells were inefficient and the induction of true cardyomyogenesis is still vigorously debated. In 1999 Makino and colleagues described the appearance of spontaneously beating MHC, MLC-2v, GATA4, Nkx2.5 positive cells following treatment of immortalized murine bone marrow stromal cells with 5′-azacytidine (5-AZA) [43]. Since then, this drug was commonly used to induce cardiomyogenesis in isolated cells [44]. Nevertheless, in some experiments several cells stained positive for adipogenic markers suggesting that this method was not cardiac selective, while other groups were unable to reproduce these findings [45]. Of note, the putative mechanism of 5-AZA induced cardiomyogenesis was firstly attributed to demethylation of cardiac-related genes. However, a study by Cho et al. [46] demonstrated that the effect of 5-AZA was not related to the epigenetic activation of cardio-specific genes, but rather to the transcriptional inhibition of the glycogen synthase kinase (GSK)-3 gene, a major player in the Wnt signaling pathway [46]. In this light, our findings suggest that the EpiC treatment determines global activation-prone changes in chromatin structure, including that at c-Kit, MDR-1 and Nkx2.5 promoters. Importantly, the expression of these genes was induced without apparently altering that of non-cardiac osteogenic and adipogenic differentiation markers. This suggests that CStC response to EpiC may be predominantly cardiovascular oriented [10], [47]. In fact, along with the expression of adult cardiac stem markers, EpiC treatment also induced the up-regulation of specific transcription factors associated with the vascular and cardiomyocyte lineage commitment.

It is widely accepted that microRNAs (miRs), are important for cardiac gene expression regulation and differentiation control [48], [49]. In the present study, miR expression has been evaluated by profiling analysis. The evidence that unsupervised cluster analysis correctly separated between EpiC-treated and control CStC, further indicates that the EpiC treatment is potentially able to induce a specific transcriptional programme. Specifically, the expression of miR-133a, associated with cardiomyogenic differentiation [50], was strongly up-regulated, together with the expression of miR-210 and mir-34a, involved in stem cell survival [51] and negative growth control [52], respectively. Of note, miR-155 whose expression is associated with proliferation [53] and to the protection of cardiomyocyte precursors from apoptosis [54], was down-modulated in agreement with the reduced proliferation ability observed in EpiC-treated CStC. Interestingly, a recent paper by Anversa and co-workers shows that microRNA typically associated to adult cardiomyocytes (such as miR-1, mir-499 and mir-133) are expressed in cardiovascular precursors, but at lower levels than in adult cardiomyocytes [49]. In this light Epic-treated CStC expressing miR-133, more closely resemble cardiovascular precursors than control CStC, in which miR-133 expression is very low.

Based on the expression of typical stem and cardiovascular precursor markers and considering previous hypothesis for cardiovascular stem cell differentiation hierarchy in the adult heart [16], it is difficult to establish the differentiation stage to which CStC may belong. The fact that untreated CStC are GATA4, GATA6 and Mef2C positive, but only EpiC-treated CStC are cKit, MDR-1and VEGFR2 positive suggests that our cocktail may induce a cascade of events able to reprogram CStC towards a more immature state, characterized by the expression of cardiovascular stem cell markers [55]. On the other hand, EpiC-treated cells also up-regulated some markers associated with differentiating cardiomyocyte precursors (i.e. Nkx2.5, Gata4, α–sarcomeric actin, α–myosin heavy chain, miR-133a) and exhibited functional, although not yet operational, properties typical of differentiating cardiomyocytes, suggesting that EpiC treatment may induce cardiomyogenic differentiation at least in a fraction of the CStC population. It is thus possible that our approach induced the production of a mixed population composed of cells at different stages of differentiation, or acted on different cell populations present inside the CStC preparations. More experiments are required to elucidate this important point.

Importantly, many cells presented a fast sodium current (normally responsible for the action potential upstroke), which, however, was small in size and with kinetic properties slightly different from those of mature channels. In fact, TTX-sensitivity and expression analyses evidenced that EpiC-treated cells expressed both the TTX-resistant NaV1.5 isoform, the primary cardiac type, and the TTX-sensitiveNaV1.2 isoform, a typical neuronal type which has been also detected, albeit at low levels, in the mouse and human hearts [56], [57].

Moreover, few cells displayed the If current, which, although negligible in the adult ventricle, is present in atria and ventricles during the late embryonic and perinatal stage [27], [58].

However, due to the lack of inwardly rectifying potassium currents, such as IK1, EpiC-treated cells exhibited a depolarized resting potential keeping both the Na+ and the HCN4 channels inactive, thus preventing the Epic-treated CStC cells from acquiring induced or spontaneous electrical activity.

In conclusion, it is reported here the first evidence that CStC may be chemically reprogrammed to acquire functionally competent cardiac precursor-like features.

## Supporting Information

Figure S1 **CStC characterization and Epigenetic Cocktail (EpiC) design.** (A) Representative FACS analysis of CStC surface markers. (B) Western blot showing MDR-1, c-Kit, and VEGFR-2 expression of CStC cultured in growth medium (GM) or in low serum (LS) with or without epigenetic drugs for 7 days. ATRA = all-trans-retinoic acid; PB = phenyl butyrate; DETA/NO = diethylenetriamine/nitric oxide; EpiC = LS+ATRA+PB+DETA/NO. (C) Real-Time RT-PCR analysis demonstrates no up-regulation of adipogenic (Adipsin and PPRγ2) and osteogenic (Osteopontin) markers (n = 3). ns = not significant.
(TIF)

Figure S2 **Effects of EpiC treatment on c-Kit and MDR-1 expression in CStC.** (A) Representative immunofluorescence images for c-Kit and MDR-1 in GM and EpiC-treated CStC. Original magnification: 20×. (B) Rhodamine 123 assay in GM and EpiC treatments (n = 4). Only EpiC-treated CStC were able to extrude Rhodamine through Verapamil sensitive MDR-1 channels.
(TIF)

Figure S3 **Hierarchical clustering of microRNAs in GM and EpiC-treated CStC.** Unsupervised cluster analysis was performed using the whole dataset of microRNAs that passed the quality assurance and filtering criteria: the global expression profile discriminates treatment groups.
(TIF)

Figure S4 **Effect of EpiC treatment on the expression of the pacemaker channel subunit HCN4 in CStC.** Representative immunofluorescence images for HCN4 in GM and EpiC-treated CStC. Original magnification: 40×.
(TIF)

Figure S5 **Effect of EpiC treatment on HDAC activity in CStC.** Bar graphs show Class I HDAC activity in CStC cultured in GM or EpiC for 7 days (n = 4; * P≤0.05).
(TIF)

Figure S6 **Effects of EpiC treatment on specific-gene promoters in CStC.** (A) and (B) Bar graphs show relative enrichment for H3KMe3 and H3S10P in Nkx2.5 and GNL3 (nucleostemin) promoter.
(TIF)

Table S1 **List and working concentrations of primary antibodies used.**
(DOC)

Table S2 **List of primers for Real-Time RT-PCR.**
(DOC)

Table S3 **microRNA normalized relative expression levels in cardiac mesenchymal stromal cells (CStC) cultured in growth medium (GM) or Epigenetic Cocktail (EpiC) are expressed as mean ± SD.** The dataset includes microRNAs that passed the quality assurance and filtering criteria.
(DOC)

Document S1 **Additional Method section.**
(DOC)
