# Supplementary figures and images for: In Vitro Epigenetic Reprogramming of Human Cardiac Mesenchymal Stromal Cells into Functionally Competent Cardiovascular Precursors
Source: PLoS One. 2012 Dec 17;7(12):e51694. doi: 10.1371/journal.pone.0051694 (PMC3524246; doi:10.1371/journal.pone.0051694)

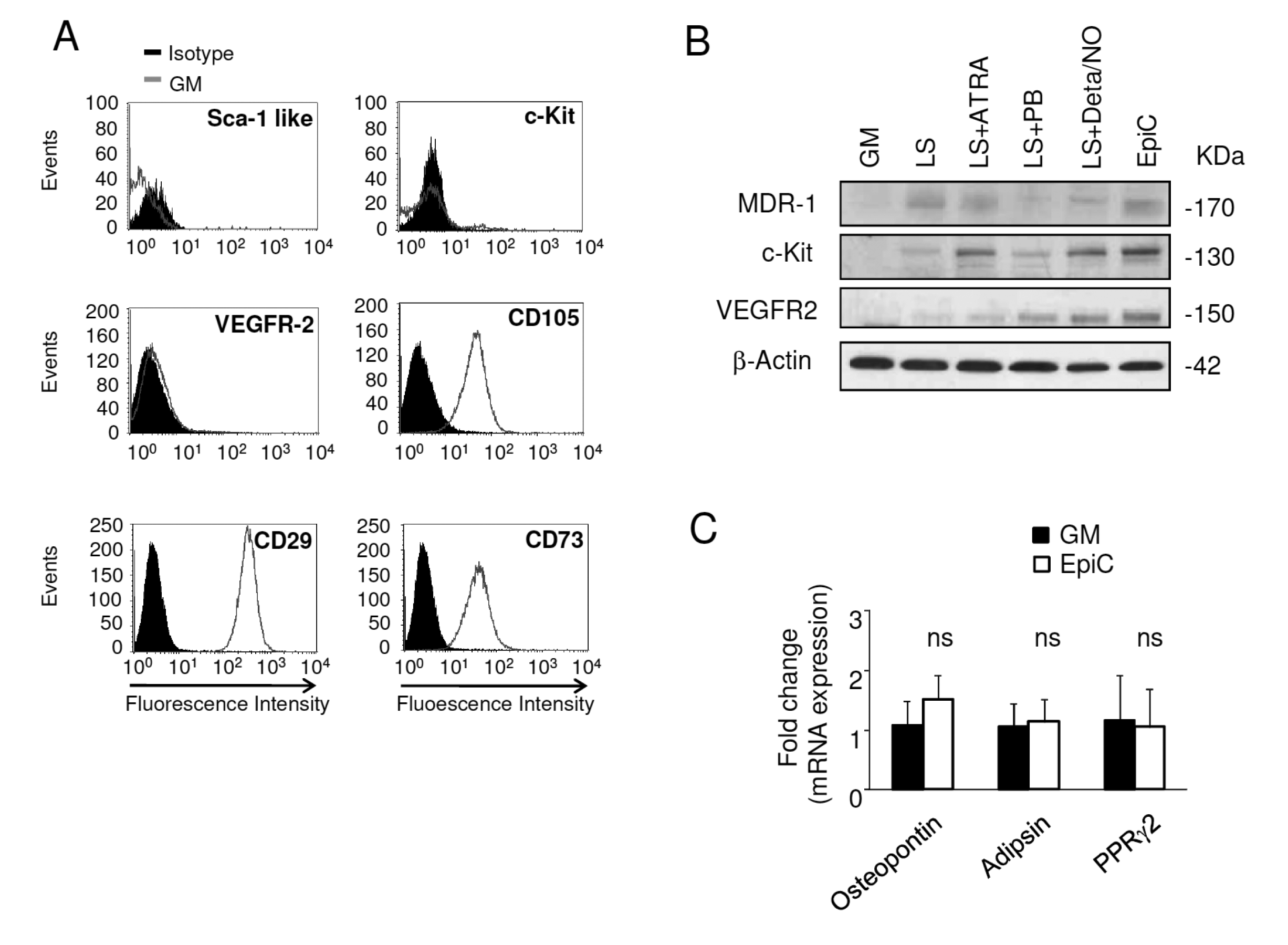

Supplement: Figure S1 — CStC characterization and Epigenetic Cocktail (EpiC) design. (A) Representative FACS analysis of CStC surface markers. (B) Western blot showing MDR-1, c-Kit, and VEGFR-2 expression of CStC cultured in growth medium (GM) or in low serum (LS) with or without epigenetic drugs for 7 days. ATRA = all-trans-retinoic acid; PB = phenyl butyrate; DETA/NO = diethylenetriamine/nitric oxide; EpiC = LS+ATRA+PB+DETA/NO. (C) Real-Time RT-PCR analysis demonstrates no up-regulation of adipogenic (Adipsin and PPRγ2) and osteogenic (Osteopontin) markers (n = 3). ns = not significant. (TIF) [file pone.0051694.s001.tif]

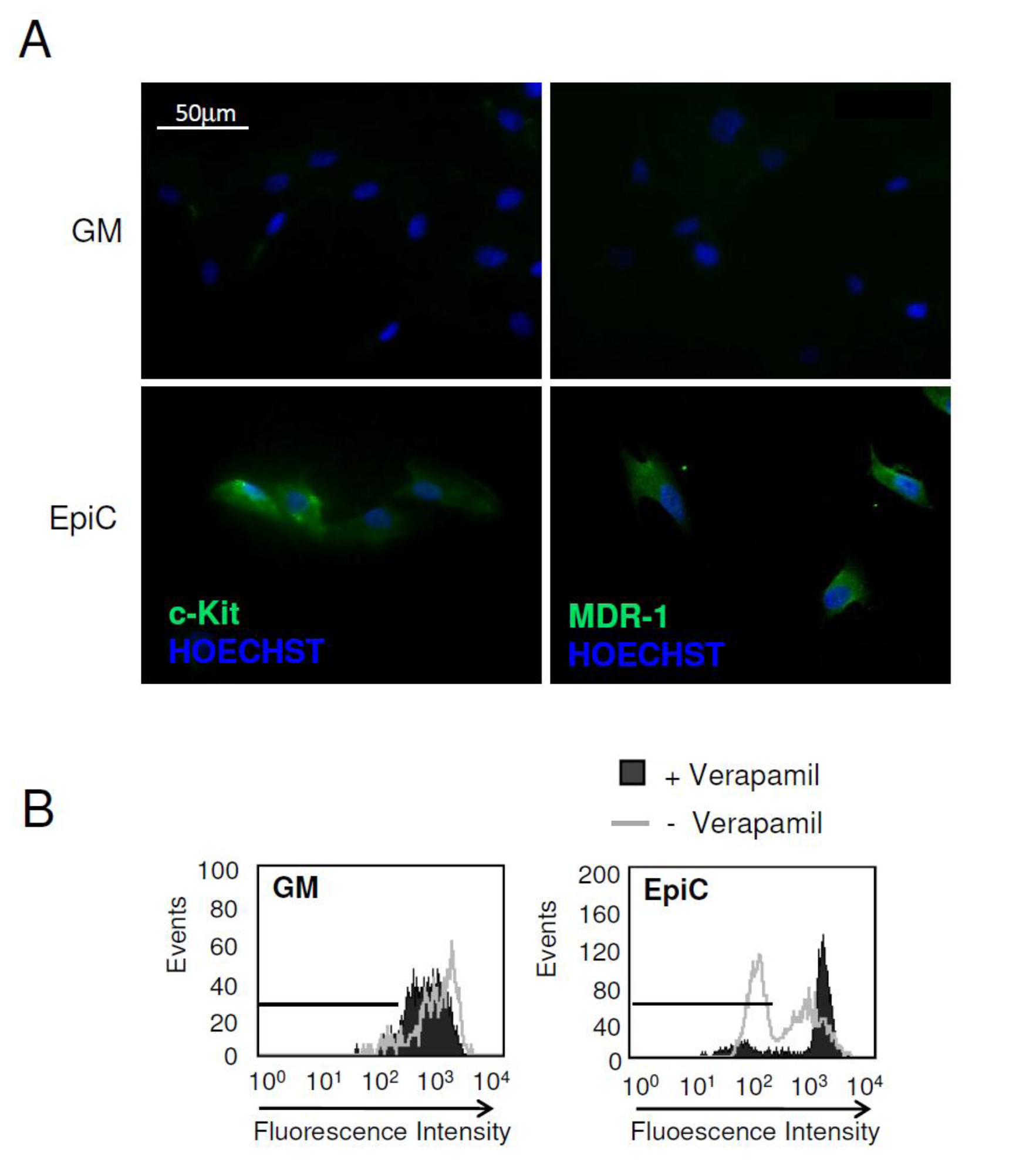

Supplement: Figure S2 — Effects of EpiC treatment on c-Kit and MDR-1 expression in CStC. (A) Representative immunofluorescence images for c-Kit and MDR-1 in GM and EpiC-treated CStC. Original magnification: 20×. (B) Rhodamine 123 assay in GM and EpiC treatments (n = 4). Only EpiC-treated CStC were able to extrude Rhodamine through Verapamil sensitive MDR-1 channels. (TIF) [file pone.0051694.s002.tif]

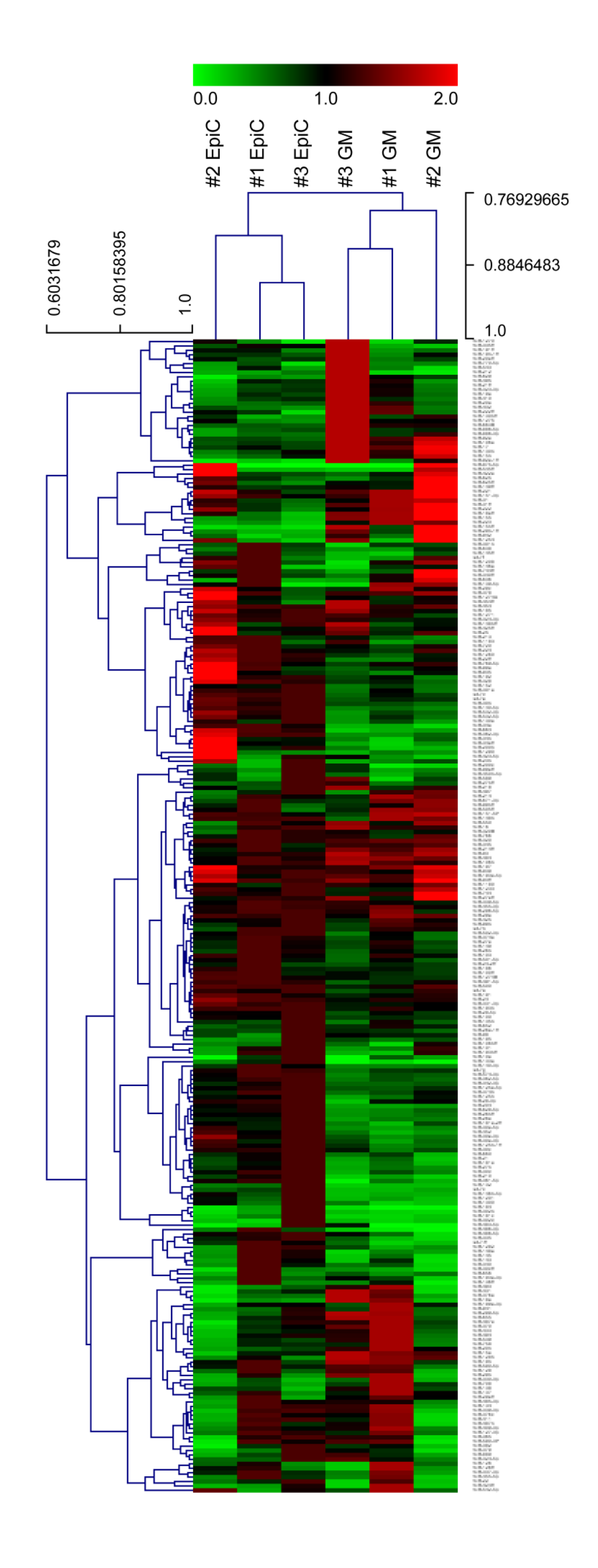

Supplement: Figure S3 — Hierarchical clustering of microRNAs in GM and EpiC-treated CStC. Unsupervised cluster analysis was performed using the whole dataset of microRNAs that passed the quality assurance and filtering criteria: the global expression profile discriminates treatment groups. (TIF) [file pone.0051694.s003.tif]

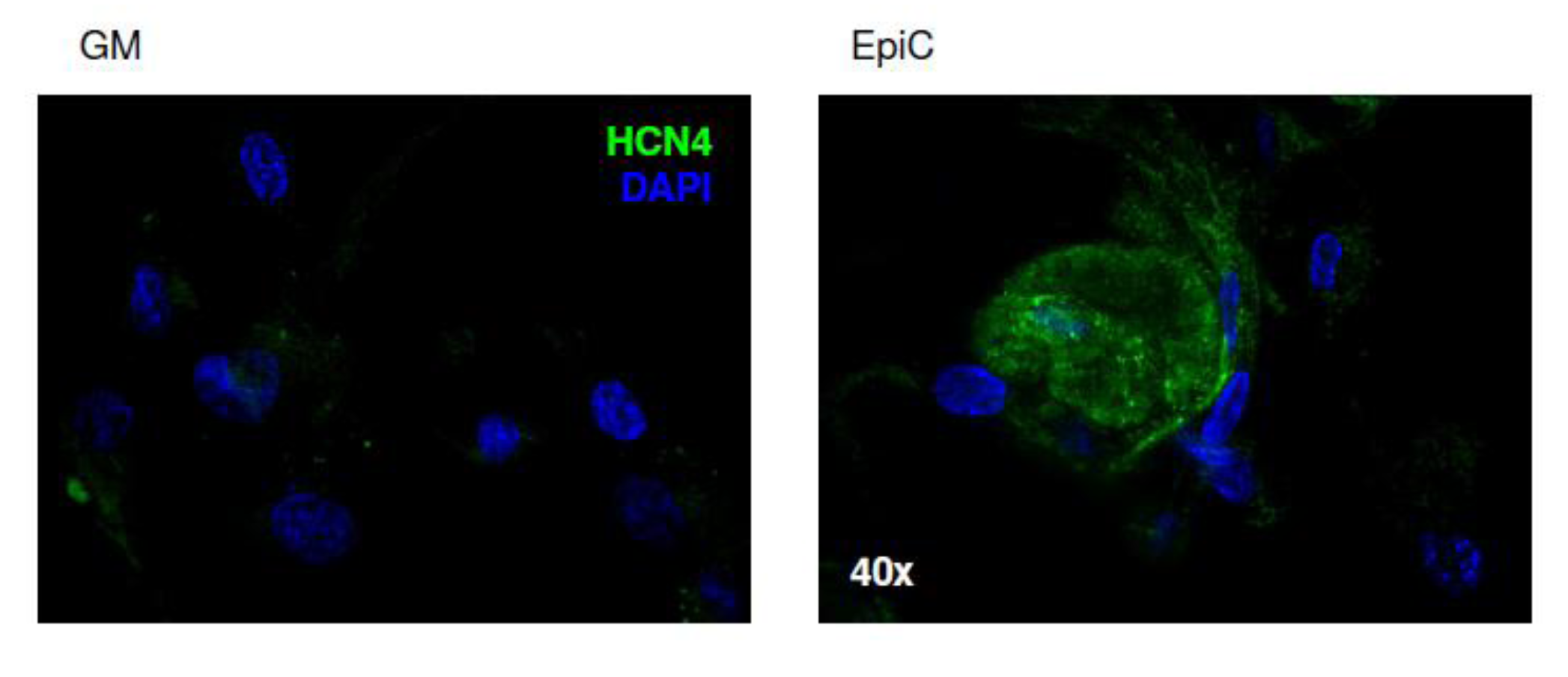

Supplement: Figure S4 — Effect of EpiC treatment on the expression of the pacemaker channel subunit HCN4 in CStC. Representative immunofluorescence images for HCN4 in GM and EpiC-treated CStC. Original magnification: 40×. (TIF) [file pone.0051694.s004.tif]

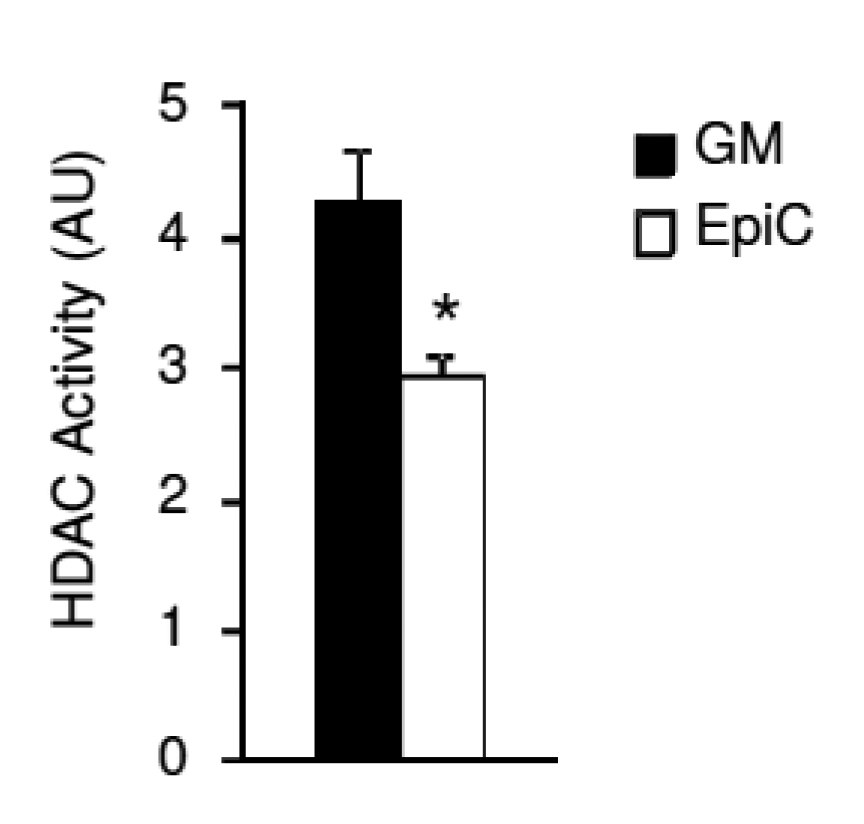

Supplement: Figure S5 — Effect of EpiC treatment on HDAC activity in CStC. Bar graphs show Class I HDAC activity in CStC cultured in GM or EpiC for 7 days (n = 4; * P≤0.05). (TIF) [file pone.0051694.s005.tif]

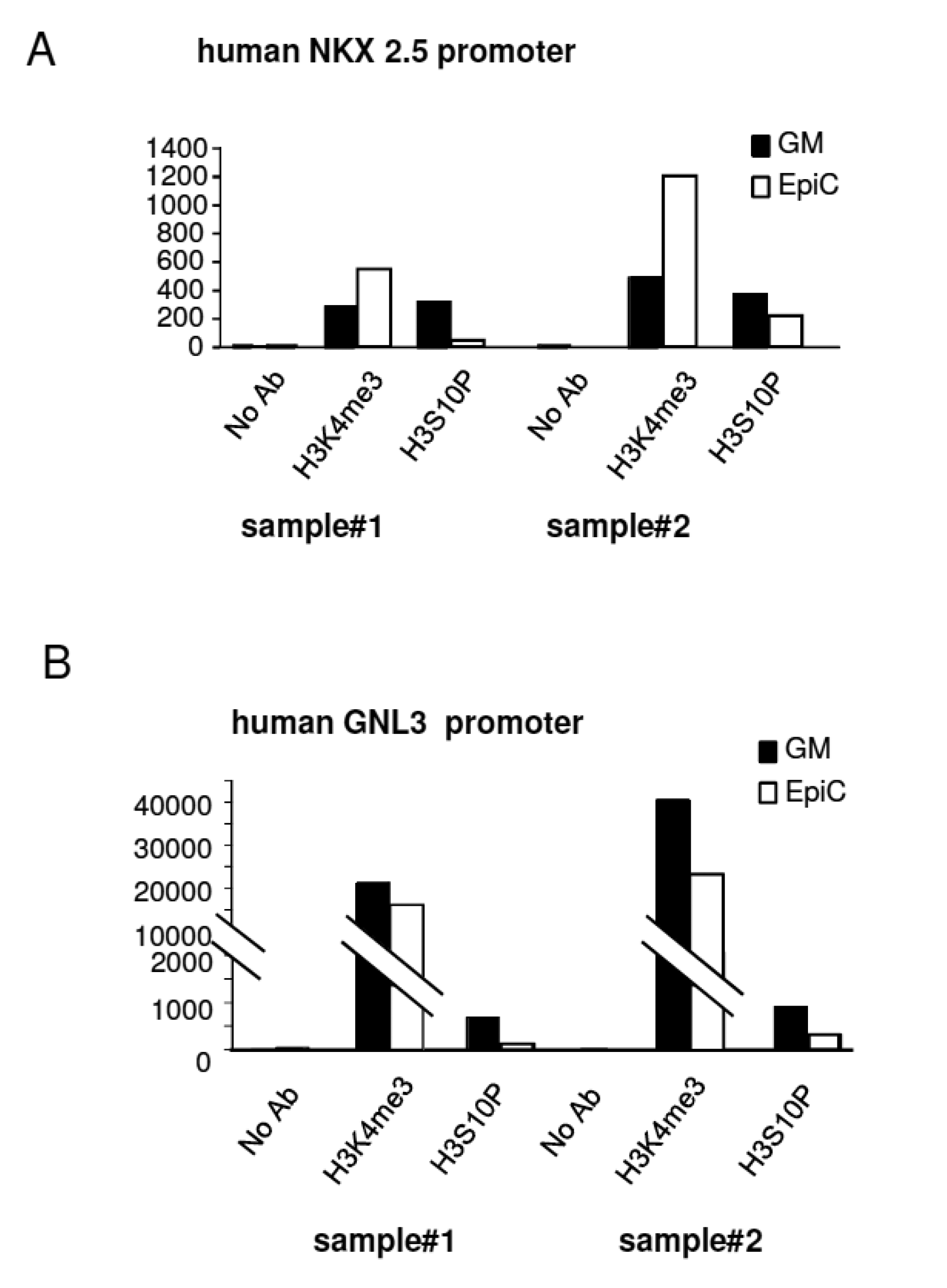

Supplement: Figure S6 — Effects of EpiC treatment on specific-gene promoters in CStC. (A) and (B) Bar graphs show relative enrichment for H3KMe3 and H3S10P in Nkx2.5 and GNL3 (nucleostemin) promoter. (TIF) [file pone.0051694.s006.tif]
