# Supplementary material for: In Vitro Epigenetic Reprogramming of Human Cardiac Mesenchymal Stromal Cells into Functionally Competent Cardiovascular Precursors
Source: PLoS One. 2012 Dec 17;7(12):e51694. doi: 10.1371/journal.pone.0051694 (PMC3524246; doi:10.1371/journal.pone.0051694)
Supplement: Table S1 — List and working concentrations of primary antibodies used. (DOC) [file pone.0051694.s007.doc]

**Table S1.**

| **Antibody** | **Manufacturer** | **Dilution** |
| --- | --- | --- |
| rabbit anti human c-Kit | DAKO | 1:200 |
| rabbit anti human VEGFR-2 | Abcam | 1:250 |
| mouse anti human MDR-1 | Sigma Aldrich | 1:250 |
| goat anti human Nucleostemin | R&D Systems | 1:500 |
| rabbit anti human H3S10P | Abcam | 1:1000 |
| rabbit anti-human Numb | Cell Signaling | 1:1000 |
| rabbit anti human Notch | Santa Cruz Biotechnology | 1:400 |
| mouse anti human Jagged | Santa Cruz Biotechnology | 1:500 |
| goat anti human GATA4 | Santa Cruz Biotechnology | 1:250 |
| mouse anti human GATA6 | Santa Cruz Biotechnology | 1:500 |
| rabbit anti human α-Smooth Muscle Actin | Santa Cruz Biotechnology | 1:500 |
| rabbit anti human Nkx2.5 | Santa Cruz Biotechnology | 1:500 |
| rabbit anti human TroponinT-C | Santa Cruz Biotechnology | 1:500 |
| mouse anti human α-Myosin Heavy Chain | Abcam | 1:500 |
| mouse anti α-Sarcomeric Actin | Sigma Aldrich | 1:250 |
| rabbit anti-HCN4 | Alomone | 1:200 |
| rabbit anti human H3K9Ac | Abcam | 1:1000 |
| rabbit anti human H3K9Me3 | Abcam | 1:500 |
| rabbit anti human H3K27Me3 | Abcam | 1:500 |
| rabbit anti human H4K16Ac | Abcam | 1:1000 |
| rabbit anti human H4K20Me | Abcam | 1:500 |
| rabbit anti human H4K20Me3 | Abcam | 1:500 |
| rabbit anti human H3S10P | Abcam | 1:500 |
| rabbit anti human H3 Total | Abcam | 1:1000 |
| rabbit anti human H4 Total | Abcam | 1:1000 |
