# Supplementary material for: In Vitro Epigenetic Reprogramming of Human Cardiac Mesenchymal Stromal Cells into Functionally Competent Cardiovascular Precursors
Source: PLoS One. 2012 Dec 17;7(12):e51694. doi: 10.1371/journal.pone.0051694 (PMC3524246; doi:10.1371/journal.pone.0051694)
Supplement: Table S2 — List of primers for Real-Time RT-PCR. (DOC) [file pone.0051694.s008.doc]

**Table S2.**

| **Primer** | **Sequence** |
| --- | --- |
| SCN5A fw | 5’-GGCCTCCTCAGCCCCATCCTC-3’ |
| SCN5A rev | 5’-GGCATCGGCAAAGTCAGACA-3’ |
| c-Kit fw | 5′-GCTTTTCTTACCAGGTGCCAAA-3 |
| c-Kit rev | 5′-GAGGATATTTCTGGCTGCCAAGT-3 |
| MDR-1 fw | 5′-GGCTCCGATACATGGTTTTCC-3 |
| MDR-1 rev | 5′-CCAGTGGTGTTTTTAGGGTCATC-3 |
| Adipsin fw | 5’-GACACCATCGACCACGACC-3’ |
| Adipsin rev | 5′- GCCACGTCGCAGAGAGTTC-3’ |
| Osteopontin fw | 5′-GCCGAGGTGATAGTGTGGTT-3’ |
| Osteopontin rev | 5′-TGAGGTGATGTCCTCGTCTG-3’ |
| PPAR-γ2 fw | 5′-TCCTTCACTGATACACTGTCTGC-3’ |
| PPAR-γ2 rev | 5′-CATTACGGAGAGATCCACGGA-3’ |
| GAPDH fw | 5′-CCACCCATGGCAAATTCC-3’ |
| GAPDH rev | 5’-TCGCTCCTGGAAGATGGTG-3’ |
