# Supplementary material for: In Vitro Epigenetic Reprogramming of Human Cardiac Mesenchymal Stromal Cells into Functionally Competent Cardiovascular Precursors
Source: PLoS One. 2012 Dec 17;7(12):e51694. doi: 10.1371/journal.pone.0051694 (PMC3524246; doi:10.1371/journal.pone.0051694)
Supplement: Table S3 — microRNA normalized relative expression levels in cardiac mesenchymal stromal cells (CStC) cultured in growth medium (GM) or Epigenetic Cocktail (EpiC) are expressed as mean ± SD. The dataset includes microRNAs that passed the quality assurance and filtering criteria. (DOC) [file pone.0051694.s009.doc]

**Table S3.**

| **microRNA** | **GM** | **EpiC** | **Fold Change** | **Raw P-value** |
| --- | --- | --- | --- | --- |
| miR-133a | 0.07 ± 0.07 | 0.86 ± 0.43 | 11.88 | 0.0488 |
| miR-184 | 0.06 ± 0.05 | 0.59 ± 0.63 | 9.34 | 0.0642 |
| miR-486-3p | 0.11 ± 0.09 | 0.83 ± 0.44 | 7.77 | 0.0534 |
| miR-34a | 0.26 ± 0.10 | 1.23 ± 0.13 | 4.78 | 0.0266 |
| miR-664 | 0.30 ± 0.09 | 1.44 ± 0.34 | 4.75 | 0.0035 |
| miR-34a# | 0.31 ± 0.13 | 1.33 ± 0.61 | 4.27 | 0.0209 |
| miR-210 | 0.25 ± 0.10 | 1.04 ± 0.29 | 4.19 | 0.0266 |
| miR-200b | 0.33 ± 0.16 | 1.34 ± 0.63 | 4.06 | 0.0492 |
| miR-361-5p | 0.30 ± 0.24 | 1.20 ± 0.11 | 3.98 | 0.1386 |
| miR-181c | 0.18 ± 0.04 | 0.66 ± 0.57 | 3.57 | 0.2559 |
| miR-146b-5p | 0.29 ± 0.04 | 1.00 ± 0.31 | 3.42 | 0.0442 |
| miR-132 | 0.24 ± 0.04 | 0.79 ± 0.44 | 3.29 | 0.1058 |
| miR-1201 | 0.30 ± 0.07 | 0.95 ± 0.35 | 3.15 | 0.0771 |
| let-7i# | 0.34 ± 0.21 | 1.05 ± 0.27 | 3.11 | 0.0953 |
| miR-335 | 0.36 ± 0.26 | 1.10 ± 0.21 | 3.10 | 0.0539 |
| miR-145 | 0.31 ± 0.23 | 0.95 ± 0.47 | 3.09 | 0.0346 |
| miR-362-3p | 0.48 ± 0.10 | 1.50 ± 0.35 | 3.09 | 0.0002 |
| miR-302b | 0.19 ± 0.05 | 0.59 ± 0.62 | 3.06 | 0.2872 |
| let-7c | 0.29 ± 0.05 | 0.89 ± 0.35 | 3.03 | 0.0682 |
| miR-1300 | 0.31 ± 0.18 | 0.93 ± 0.38 | 3.02 | 0.0126 |
| miR-204 | 0.38 ± 0.03 | 1.14 ± 0.16 | 2.99 | 0.0124 |
| miR-34b | 0.44 ± 0.23 | 1.28 ± 0.65 | 2.92 | 0.0516 |
| miR-452 | 0.42 ± 0.13 | 1.20 ± 0.17 | 2.87 | 0.0113 |
| miR-148a | 0.33 ± 0.16 | 0.95 ± 0.48 | 2.83 | 0.1923 |
| miR-30a-5p | 0.43 ± 0.05 | 1.13 ± 0.23 | 2.63 | 0.0056 |
| miR-21 | 0.40 ± 0.19 | 1.02 ± 0.29 | 2.52 | 0.0478 |
| miR-181a | 0.39 ± 0.15 | 0.98 ± 0.30 | 2.52 | 0.0627 |
| miR-302d | 0.23 ± 0.05 | 0.56 ± 0.64 | 2.47 | 0.4742 |
| miR-140-5p | 0.52 ± 0.07 | 1.28 ± 0.05 | 2.46 | 0.0114 |
| miR-1290 | 0.41 ± 0.10 | 1.00 ± 0.90 | 2.42 | 0.2960 |
| miR-30e-3p | 0.55 ± 0.12 | 1.31 ± 0.27 | 2.37 | 0.0181 |
| miR-532-3p | 0.58 ± 0.17 | 1.36 ± 0.23 | 2.32 | 0.0560 |
| miR-30d | 0.50 ± 0.20 | 1.16 ± 0.19 | 2.32 | 0.0869 |
| miR-145# | 0.39 ± 0.26 | 0.89 ± 0.38 | 2.32 | 0.2226 |
| miR-27b | 0.45 ± 0.10 | 1.03 ± 0.27 | 2.31 | 0.0758 |
| miR-143 | 0.40 ± 0.40 | 0.91 ± 0.49 | 2.25 | 0.0859 |
| miR-26b# | 0.49 ± 0.07 | 1.09 ± 0.18 | 2.22 | 0.0423 |
| miR-628-5p | 0.50 ± 0.11 | 1.12 ± 0.18 | 2.22 | 0.0657 |
| miR-26a | 0.47 ± 0.09 | 1.03 ± 0.23 | 2.19 | 0.0814 |
| miR-22# | 0.64 ± 0.25 | 1.40 ± 0.31 | 2.19 | 0.0162 |
| miR-423-5p | 0.55 ± 0.43 | 1.20 ± 0.83 | 2.17 | 0.4166 |
| miR-30d# | 0.30 ± 0.14 | 0.65 ± 0.61 | 2.15 | 0.4198 |
| miR-23b | 0.50 ± 0.19 | 1.08 ± 0.73 | 2.14 | 0.4519 |
| miR-181a-2# | 0.49 ± 0.23 | 1.05 ± 0.22 | 2.13 | 0.0919 |
| miR-140-3p | 0.49 ± 0.02 | 1.03 ± 0.28 | 2.12 | 0.0538 |
| miR-532-5p | 0.61 ± 0.21 | 1.28 ± 0.20 | 2.11 | 0.1040 |
| miR-130a | 0.51 ± 0.18 | 1.07 ± 0.28 | 2.09 | 0.1673 |
| miR-486-5p | 0.56 ± 0.42 | 1.17 ± 0.12 | 2.09 | 0.1910 |
| miR-10a | 0.33 ± 0.31 | 0.69 ± 0.64 | 2.08 | 0.8179 |
| miR-30a-3p | 0.56 ± 0.19 | 1.14 ± 0.25 | 2.03 | 0.0140 |
| miR-192 | 0.73 ± 0.23 | 1.47 ± 0.49 | 2.03 | 0.1898 |
| miR-574-3p | 0.60 ± 0.11 | 1.21 ± 0.16 | 2.02 | 0.0445 |
| miR-660 | 0.53 ± 0.25 | 1.06 ± 0.24 | 2.00 | 0.0858 |
| miR-1282 | 0.49 ± 0.21 | 0.98 ± 0.52 | 2.00 | 0.3107 |
| miR-200c | 0.36 ± 0.41 | 0.71 ± 0.53 | 1.99 | 0.0643 |
| miR-362-5p | 0.60 ± 0.08 | 1.17 ± 0.18 | 1.94 | 0.0349 |
| miR-328 | 0.76 ± 0.15 | 1.46 ± 0.38 | 1.93 | 0.1235 |
| miR-125b-1# | 0.62 ± 0.17 | 1.19 ± 0.14 | 1.92 | 0.0221 |
| miR-30b | 0.66 ± 0.07 | 1.27 ± 0.14 | 1.92 | 0.0345 |
| miR-340 | 0.46 ± 0.26 | 0.88 ± 0.45 | 1.91 | 0.0223 |
| miR-935 | 0.68 ± 0.24 | 1.29 ± 0.65 | 1.88 | 0.0827 |
| miR-1260 | 0.77 ± 0.15 | 1.38 ± 0.22 | 1.80 | 0.0285 |
| miR-125b | 0.61 ± 0.29 | 1.08 ± 0.19 | 1.76 | 0.2314 |
| miR-152 | 0.62 ± 0.14 | 1.08 ± 0.19 | 1.75 | 0.1176 |
| miR-301a | 0.70 ± 0.27 | 1.22 ± 0.08 | 1.75 | 0.1292 |
| miR-502-3p | 0.69 ± 0.27 | 1.19 ± 0.18 | 1.73 | 0.0993 |
| let-7g | 0.71 ± 0.14 | 1.22 ± 0.12 | 1.72 | 0.0672 |
| miR-342-3p | 0.62 ± 0.04 | 1.07 ± 0.24 | 1.71 | 0.0825 |
| miR-301b | 0.54 ± 0.34 | 0.92 ± 0.35 | 1.69 | 0.2902 |
| let-7d | 0.77 ± 0.18 | 1.29 ± 0.03 | 1.69 | 0.0639 |
| miR-99a | 0.81 ± 0.21 | 1.37 ± 0.65 | 1.68 | 0.0764 |
| miR-450b-5p | 0.49 ± 0.17 | 0.82 ± 0.49 | 1.68 | 0.1779 |
| miR-193b# | 0.57 ± 0.53 | 0.96 ± 0.53 | 1.68 | 0.5626 |
| let-7e | 0.78 ± 0.11 | 1.30 ± 0.11 | 1.68 | 0.0404 |
| miR-30c | 0.72 ± 0.20 | 1.20 ± 0.09 | 1.68 | 0.0649 |
| miR-28-3p | 0.73 ± 0.43 | 1.22 ± 0.08 | 1.68 | 0.3034 |
| miR-213 | 0.68 ± 0.23 | 1.14 ± 0.31 | 1.67 | 0.2820 |
| miR-483-5p | 0.32 ± 0.51 | 0.53 ± 0.67 | 1.62 | 0.2387 |
| miR-125a-5p | 0.72 ± 0.10 | 1.16 ± 0.14 | 1.62 | 0.0736 |
| miR-22 | 0.58 ± 0.63 | 0.89 ± 0.38 | 1.55 | 0.3833 |
| miR-374b | 0.74 ± 0.21 | 1.15 ± 0.25 | 1.55 | 0.2635 |
| miR-146a | 0.63 ± 0.41 | 0.97 ± 0.66 | 1.54 | 0.0200 |
| miR-374a | 0.74 ± 0.22 | 1.13 ± 0.19 | 1.54 | 0.0256 |
| miR-638 | 0.55 ± 0.26 | 0.84 ± 0.43 | 1.54 | 0.4966 |
| miR-501-5p | 0.73 ± 0.21 | 1.10 ± 0.20 | 1.51 | 0.0199 |
| let-7f | 0.67 ± 0.58 | 1.00 ± 0.44 | 1.50 | 0.2731 |
| miR-491-5p | 0.83 ± 0.23 | 1.24 ± 0.08 | 1.49 | 0.1071 |
| miR-99a# | 0.56 ± 0.25 | 0.82 ± 0.44 | 1.47 | 0.0294 |
| miR-1183 | 0.73 ± 0.47 | 1.07 ± 0.43 | 1.47 | 0.5530 |
| miR-1274B | 0.86 ± 0.09 | 1.24 ± 0.15 | 1.44 | 0.1077 |
| miR-136# | 0.48 ± 0.42 | 0.70 ± 0.55 | 1.44 | 0.3161 |
| miR-769-5p | 1.00 ± 0.24 | 1.44 ± 0.53 | 1.43 | 0.0707 |
| miR-100 | 0.80 ± 0.04 | 1.12 ± 0.16 | 1.41 | 0.0925 |
| miR-28-5p | 0.80 ± 0.15 | 1.12 ± 0.15 | 1.40 | 0.1875 |
| miR-331-3p | 0.94 ± 0.06 | 1.30 ± 0.11 | 1.38 | 0.0116 |
| miR-224 | 1.00 ± 0.28 | 1.37 ± 0.33 | 1.37 | 0.1159 |
| miR-100# | 0.85 ± 0.10 | 1.16 ± 0.27 | 1.36 | 0.2888 |
| miR-26b | 0.78 ± 0.19 | 1.06 ± 0.30 | 1.36 | 0.2450 |
| miR-186 | 0.83 ± 0.09 | 1.12 ± 0.22 | 1.35 | 0.1271 |
| miR-324-3p | 0.99 ± 0.16 | 1.34 ± 0.19 | 1.35 | 0.1288 |
| let-7a | 0.89 ± 0.27 | 1.18 ± 0.12 | 1.33 | 0.2533 |
| miR-103 | 0.91 ± 0.31 | 1.19 ± 0.15 | 1.31 | 0.1548 |
| miR-101 | 0.71 ± 0.45 | 0.93 ± 0.49 | 1.31 | 0.7553 |
| miR-720 | 0.93 ± 0.20 | 1.22 ± 0.32 | 1.30 | 0.3630 |
| miR-149 | 0.83 ± 0.24 | 1.07 ± 0.24 | 1.30 | 0.0489 |
| miR-337-3p | 0.79 ± 0.61 | 1.01 ± 0.27 | 1.28 | 0.2783 |
| miR-193a-3p | 0.63 ± 0.41 | 0.80 ± 0.46 | 1.28 | 0.4886 |
| miR-191 | 0.99 ± 0.15 | 1.26 ± 0.16 | 1.28 | 0.0221 |
| miR-656 | 0.54 ± 0.12 | 0.69 ± 0.55 | 1.27 | 0.9285 |
| miR-24-2# | 0.76 ± 0.10 | 0.97 ± 0.32 | 1.27 | 0.1940 |
| miR-106b# | 0.61 ± 0.48 | 0.77 ± 0.46 | 1.27 | 0.5844 |
| miR-24 | 1.09 ± 0.11 | 1.38 ± 0.18 | 1.27 | 0.0590 |
| miR-1274A | 0.96 ± 0.13 | 1.20 ± 0.74 | 1.26 | 0.7573 |
| miR-1208 | 0.94 ± 0.76 | 1.17 ± 0.45 | 1.25 | 0.3827 |
| miR-193b | 1.03 ± 0.15 | 1.28 ± 0.03 | 1.24 | 0.1177 |
| miR-135b | 0.75 ± 0.30 | 0.92 ± 0.33 | 1.24 | 0.6348 |
| miR-1271 | 1.04 ± 0.29 | 1.28 ± 0.15 | 1.23 | 0.4200 |
| miR-27a | 0.88 ± 0.25 | 1.08 ± 0.24 | 1.23 | 0.1292 |
| miR-545# | 0.71 ± 0.88 | 0.86 ± 1.01 | 1.22 | 0.0364 |
| miR-335# | 0.75 ± 0.84 | 0.91 ± 0.21 | 1.22 | 0.4038 |
| miR-455-5p | 0.71 ± 0.65 | 0.87 ± 0.43 | 1.22 | 0.2602 |
| miR-26a-1# | 0.75 ± 0.13 | 0.91 ± 0.34 | 1.20 | 0.4412 |
| miR-500 | 1.09 ± 0.24 | 1.31 ± 0.03 | 1.20 | 0.2335 |
| miR-425# | 1.10 ± 0.37 | 1.31 ± 0.63 | 1.18 | 0.7359 |
| miR-185 | 1.22 ± 0.33 | 1.39 ± 0.36 | 1.14 | 0.6599 |
| miR-339-5p | 1.05 ± 0.15 | 1.19 ± 0.17 | 1.14 | 0.1491 |
| miR-126# | 0.76 ± 0.78 | 0.86 ± 0.35 | 1.13 | 0.3791 |
| miR-411 | 0.85 ± 0.69 | 0.96 ± 0.56 | 1.12 | 0.3673 |
| miR-148b# | 1.10 ± 0.55 | 1.22 ± 0.50 | 1.11 | 0.6871 |
| miR-939 | 1.32 ± 0.39 | 1.44 ± 0.38 | 1.09 | 0.0892 |
| let-7b | 1.00 ± 0.23 | 1.09 ± 0.35 | 1.09 | 0.8546 |
| miR-195 | 0.65 ± 0.10 | 0.71 ± 0.51 | 1.09 | 0.8591 |
| miR-382 | 0.67 ± 0.37 | 0.72 ± 0.62 | 1.09 | 0.5859 |
| miR-589 | 0.78 ± 0.65 | 0.85 ± 0.45 | 1.08 | 0.4293 |
| miR-296-5p | 1.14 ± 0.37 | 1.23 ± 0.13 | 1.08 | 0.5171 |
| miR-379 | 0.64 ± 0.44 | 0.69 ± 0.54 | 1.07 | 0.6867 |
| miR-25 | 1.21 ± 0.29 | 1.29 ± 0.66 | 1.07 | 0.9826 |
| miR-320B | 0.94 ± 0.14 | 1.01 ± 0.42 | 1.07 | 0.9961 |
| miR-127-3p | 0.95 ± 0.47 | 1.00 ± 0.42 | 1.05 | 0.7256 |
| miR-27b# | 0.80 ± 0.36 | 0.84 ± 0.40 | 1.05 | 0.7617 |
| miR-365 | 0.83 ± 0.59 | 0.87 ± 0.61 | 1.05 | 0.3107 |
| miR-425 | 1.08 ± 0.26 | 1.12 ± 0.15 | 1.04 | 0.8085 |
| miR-1233 | 1.15 ± 0.50 | 1.19 ± 0.17 | 1.04 | 0.7203 |
| miR-422a | 1.13 ± 0.68 | 1.17 ± 0.75 | 1.04 | 0.8304 |
| miR-766 | 1.10 ± 0.30 | 1.13 ± 0.21 | 1.03 | 0.8817 |
| miR-148b | 1.23 ± 0.57 | 1.27 ± 0.02 | 1.03 | 0.7484 |
| miR-485-3p | 0.89 ± 0.37 | 0.91 ± 0.51 | 1.02 | 0.7243 |
| miR-376c | 0.89 ± 0.69 | 0.90 ± 0.52 | 1.01 | 0.4979 |
| miR-455-3p | 1.22 ± 0.20 | 1.22 ± 0.11 | -1.00 | 0.9814 |
| miR-99b | 1.06 ± 0.30 | 1.05 ± 0.26 | -1.02 | 0.9861 |
| miR-29c | 1.18 ± 0.43 | 1.16 ± 0.57 | -1.02 | 0.8557 |
| miR-378 | 1.38 ± 0.20 | 1.33 ± 0.69 | -1.04 | 0.6637 |
| miR-590-5p | 1.08 ± 0.48 | 1.04 ± 0.25 | -1.04 | 0.9158 |
| miR-454 | 1.42 ± 0.28 | 1.37 ± 0.19 | -1.04 | 0.8561 |
| miR-652 | 0.93 ± 0.15 | 0.89 ± 0.36 | -1.05 | 0.7704 |
| miR-889 | 0.82 ± 0.45 | 0.78 ± 0.49 | -1.06 | 0.2599 |
| miR-29a | 1.34 ± 0.28 | 1.26 ± 0.06 | -1.06 | 0.7937 |
| miR-542-5p | 0.98 ± 0.58 | 0.93 ± 0.54 | -1.06 | 0.8933 |
| miR-339-3p | 1.00 ± 0.68 | 0.93 ± 0.59 | -1.07 | 0.7493 |
| miR-197 | 1.54 ± 0.35 | 1.42 ± 0.54 | -1.09 | 0.4152 |
| miR-134 | 1.06 ± 0.62 | 0.98 ± 0.52 | -1.09 | 0.4376 |
| miR-487b | 0.93 ± 0.69 | 0.84 ± 0.47 | -1.10 | 0.7659 |
| miR-320 | 1.38 ± 0.08 | 1.24 ± 0.08 | -1.11 | 0.0593 |
| miR-324-5p | 0.94 ± 0.44 | 0.84 ± 0.40 | -1.12 | 0.6147 |
| miR-875-5p | 0.76 ± 1.32 | 0.68 ± 1.18 | -1.12 | 0.8231 |
| miR-193a-5p | 1.48 ± 0.29 | 1.31 ± 0.37 | -1.12 | 0.2232 |
| miR-590-3P | 0.93 ± 0.72 | 0.83 ± 0.65 | -1.13 | 0.0997 |
| miR-98 | 0.92 ± 0.06 | 0.81 ± 0.46 | -1.14 | 0.5260 |
| miR-1180 | 1.22 ± 0.24 | 1.07 ± 0.26 | -1.14 | 0.3582 |
| miR-126 | 0.82 ± 0.75 | 0.72 ± 0.35 | -1.14 | 0.7503 |
| miR-744# | 1.06 ± 1.02 | 0.92 ± 0.36 | -1.15 | 0.8453 |
| miR-191# | 0.90 ± 0.71 | 0.78 ± 0.27 | -1.15 | 0.9928 |
| miR-128 | 0.97 ± 0.59 | 0.84 ± 0.42 | -1.15 | 0.9685 |
| miR-424# | 0.68 ± 0.84 | 0.59 ± 0.45 | -1.16 | 0.8383 |
| miR-93# | 1.72 ± 0.56 | 1.48 ± 0.35 | -1.16 | 0.2870 |
| miR-497 | 1.03 ± 0.53 | 0.89 ± 0.40 | -1.16 | 0.8951 |
| miR-345 | 1.26 ± 0.07 | 1.07 ± 0.24 | -1.17 | 0.3449 |
| miR-106b | 1.39 ± 0.26 | 1.18 ± 0.16 | -1.18 | 0.2309 |
| miR-151-5P | 1.38 ± 0.55 | 1.17 ± 0.18 | -1.18 | 0.6311 |
| miR-744 | 1.41 ± 0.66 | 1.20 ± 0.11 | -1.18 | 0.7639 |
| miR-454# | 1.21 ± 0.45 | 1.00 ± 0.62 | -1.22 | 0.6472 |
| miR-218 | 1.20 ± 0.42 | 0.99 ± 0.32 | -1.22 | 0.0074 |
| miR-409-3p | 1.08 ± 0.58 | 0.88 ± 0.39 | -1.22 | 0.1550 |
| miR-99b# | 1.15 ± 0.38 | 0.93 ± 0.37 | -1.23 | 0.4358 |
| miR-20a# | 1.00 ± 0.63 | 0.81 ± 0.14 | -1.24 | 0.8008 |
| miR-340# | 1.32 ± 0.93 | 1.04 ± 0.29 | -1.26 | 0.8467 |
| miR-16 | 1.33 ± 0.27 | 1.03 ± 0.36 | -1.29 | 0.2688 |
| miR-625 | 1.21 ± 1.03 | 0.92 ± 0.40 | -1.31 | 0.7722 |
| miR-671-3p | 1.26 ± 0.43 | 0.95 ± 0.33 | -1.32 | 0.4195 |
| miR-19b | 1.32 ± 0.48 | 1.00 ± 0.30 | -1.32 | 0.4882 |
| miR-484 | 1.54 ± 0.11 | 1.15 ± 0.16 | -1.34 | 0.0447 |
| miR-151-3p | 1.74 ± 0.58 | 1.28 ± 0.31 | -1.36 | 0.0345 |
| miR-214 | 1.30 ± 0.42 | 0.95 ± 0.35 | -1.37 | 0.4247 |
| miR-221 | 1.66 ± 0.34 | 1.21 ± 0.53 | -1.38 | 0.1228 |
| miR-941 | 0.99 ± 0.58 | 0.72 ± 0.37 | -1.39 | 0.5508 |
| miR-505# | 1.33 ± 0.41 | 0.96 ± 0.41 | -1.39 | 0.0562 |
| miR-199a-3p | 1.08 ± 0.51 | 0.78 ± 0.39 | -1.39 | 0.4612 |
| miR-19b-1# | 1.04 ± 0.63 | 0.75 ± 0.08 | -1.39 | 0.7012 |
| miR-139-5p | 1.10 ± 0.84 | 0.79 ± 0.64 | -1.39 | 0.0603 |
| miR-214# | 1.46 ± 0.17 | 1.04 ± 0.25 | -1.40 | 0.2151 |
| miR-543 | 0.78 ± 0.83 | 0.55 ± 0.32 | -1.41 | 0.9612 |
| miR-494 | 1.11 ± 0.56 | 0.77 ± 0.46 | -1.44 | 0.1759 |
| miR-27a# | 1.61 ± 0.74 | 1.10 ± 0.16 | -1.46 | 0.4865 |
| miR-550 | 1.37 ± 0.39 | 0.94 ± 0.45 | -1.47 | 0.4424 |
| miR-758 | 1.00 ± 0.60 | 0.67 ± 0.31 | -1.48 | 0.1791 |
| miR-93 | 1.60 ± 0.17 | 1.08 ± 0.13 | -1.49 | 0.0673 |
| miR-410 | 1.03 ± 0.62 | 0.68 ± 0.21 | -1.51 | 0.2160 |
| miR-149# | 1.44 ± 0.81 | 0.93 ± 0.21 | -1.55 | 0.1887 |
| miR-137 | 1.19 ± 0.41 | 0.75 ± 0.39 | -1.58 | 0.0434 |
| miR-212 | 0.65 ± 0.92 | 0.40 ± 0.11 | -1.64 | 0.7224 |
| miR-29a# | 1.12 ± 0.22 | 0.68 ± 0.56 | -1.64 | 0.1726 |
| miR-495 | 1.09 ± 0.65 | 0.65 ± 0.36 | -1.67 | 0.0786 |
| miR-1270 | 0.91 ± 0.78 | 0.54 ± 0.49 | -1.67 | 0.6991 |
| miR-636 | 1.10 ± 0.76 | 0.64 ± 0.59 | -1.71 | 0.4494 |
| miR-625# | 1.41 ± 0.84 | 0.83 ± 0.55 | -1.71 | 0.0372 |
| miR-31 | 1.46 ± 0.42 | 0.85 ± 0.30 | -1.73 | 0.0350 |
| miR-433 | 1.13 ± 0.52 | 0.65 ± 0.35 | -1.73 | 0.0987 |
| miR-487a | 1.19 ± 0.45 | 0.68 ± 0.54 | -1.74 | 0.2558 |
| miR-19a | 1.12 ± 0.57 | 0.64 ± 0.24 | -1.74 | 0.1076 |
| miR-655 | 1.32 ± 0.58 | 0.76 ± 0.40 | -1.75 | 0.0228 |
| miR-323-3p | 1.11 ± 0.59 | 0.63 ± 0.24 | -1.75 | 0.0482 |
| miR-539 | 1.11 ± 0.58 | 0.63 ± 0.29 | -1.75 | 0.0094 |
| miR-222 | 1.68 ± 0.68 | 0.96 ± 0.56 | -1.76 | 0.0286 |
| miR-29b | 1.14 ± 0.77 | 0.63 ± 0.46 | -1.81 | 0.3929 |
| miR-21# | 1.09 ± 0.60 | 0.59 ± 0.26 | -1.84 | 0.0355 |
| miR-299-5p | 1.30 ± 0.64 | 0.71 ± 0.42 | -1.84 | 0.0681 |
| miR-370 | 1.24 ± 0.50 | 0.67 ± 0.42 | -1.85 | 0.1059 |
| miR-1275 | 1.23 ± 0.42 | 0.64 ± 0.34 | -1.92 | 0.2335 |
| miR-222# | 1.22 ± 0.61 | 0.63 ± 0.43 | -1.94 | 0.4492 |
| miR-31# | 1.70 ± 0.38 | 0.87 ± 0.36 | -1.96 | 0.0239 |
| miR-886-5p | 1.12 ± 0.52 | 0.56 ± 0.18 | -1.99 | 0.0967 |
| miR-431 | 1.10 ± 0.84 | 0.55 ± 0.41 | -2.00 | 0.0189 |
| miR-330-3p | 0.85 ± 0.71 | 0.43 ± 0.24 | -2.01 | 0.1657 |
| miR-138 | 1.16 ± 0.47 | 0.58 ± 0.28 | -2.01 | 0.2225 |
| miR-1254 | 1.38 ± 1.07 | 0.68 ± 0.70 | -2.03 | 0.4335 |
| miR-17 | 1.68 ± 0.31 | 0.81 ± 0.13 | -2.07 | 0.0630 |
| miR-663B | 1.32 ± 0.34 | 0.63 ± 0.02 | -2.08 | 0.0397 |
| miR-20a | 1.17 ± 0.56 | 0.55 ± 0.09 | -2.11 | 0.0883 |
| miR-130b | 1.61 ± 0.39 | 0.75 ± 0.27 | -2.16 | 0.1111 |
| miR-432 | 1.20 ± 0.61 | 0.55 ± 0.15 | -2.20 | 0.3082 |
| miR-106a | 1.59 ± 0.37 | 0.72 ± 0.07 | -2.20 | 0.0603 |
| miR-20b | 1.28 ± 0.34 | 0.58 ± 0.20 | -2.21 | 0.0455 |
| miR-770-5p | 0.89 ± 0.71 | 0.40 ± 0.29 | -2.22 | 0.4659 |
| miR-15a | 1.50 ± 0.21 | 0.67 ± 0.38 | -2.25 | 0.1285 |
| miR-130b# | 1.20 ± 0.51 | 0.52 ± 0.27 | -2.31 | 0.0924 |
| miR-708 | 1.07 ± 0.51 | 0.46 ± 0.19 | -2.34 | 0.0020 |
| miR-92a | 1.58 ± 0.27 | 0.68 ± 0.07 | -2.34 | 0.0303 |
| miR-1285 | 1.54 ± 0.17 | 0.65 ± 0.36 | -2.37 | 0.0883 |
| miR-15b | 1.55 ± 0.35 | 0.64 ± 0.26 | -2.43 | 0.0831 |
| miR-886-3p | 1.24 ± 0.47 | 0.50 ± 0.12 | -2.49 | 0.0993 |
| miR-493 | 0.99 ± 0.76 | 0.38 ± 0.28 | -2.57 | 0.0789 |
| miR-376a | 1.15 ± 0.70 | 0.43 ± 0.23 | -2.71 | 0.0093 |
| miR-18a# | 1.80 ± 0.39 | 0.65 ± 0.48 | -2.76 | 0.0759 |
| miR-629 | 1.15 ± 0.50 | 0.30 ± 0.21 | -3.82 | 0.0877 |
| miR-942 | 1.38 ± 0.92 | 0.36 ± 0.22 | -3.82 | 0.0009 |
| miR-18a | 1.16 ± 0.79 | 0.30 ± 0.19 | -3.91 | 0.0072 |
| miR-223 | 1.35 ± 0.38 | 0.32 ± 0.12 | -4.17 | 0.0017 |
| miR-29b-1# | 1.51 ± 0.82 | 0.35 ± 0.09 | -4.38 | 0.0282 |
| miR-155 | 1.83 ± 0.43 | 0.40 ± 0.15 | -4.54 | 0.0206 |
| miR-92a-1# | 1.09 ± 0.64 | 0.14 ± 0.07 | -7.78 | 0.0958 |
| miR-15b# | 1.53 ± 0.75 | 0.15 ± 0.04 | -10.42 | 0.0429 |
