# Supplementary material for: In Vitro Epigenetic Reprogramming of Human Cardiac Mesenchymal Stromal Cells into Functionally Competent Cardiovascular Precursors
Source: PLoS One. 2012 Dec 17;7(12):e51694. doi: 10.1371/journal.pone.0051694 (PMC3524246; doi:10.1371/journal.pone.0051694)
Supplement: Document S1 — Additional Method section. (DOC) [file pone.0051694.s010.doc]

**Document S1**

**S1.1 Human bone marrow stromal cell (BMStC) isolation and culture**

BMStC isolation and culture was performed as previously described [1].

**S1.2 Flow citometry**

Cells were detached with 0.02% EDTA solution (Sigma-Aldrich), stained with FITC-conjugated anti-Sca-1 (BD Bioscience) and APC-conjugated anti-c-Kit CD117 (R&D) for 10 min at room temperature. Cells were then analysed using a FACSCalibur flow cytometer (BD Biosciences) equipped with Cell-Quest Software.

**S1.3 Apoptosis detection**

Apoptosis was estimated using the Cell Death Detection Elisa Plus Kit (Roche), following manufacturer’s instructions.

**S1.4 β-Galactosidase acid assay**

To evaluate senescence, β-galactosidase acid staining was performed according to the manufacturer’s instructions (Cell Signalling Technology).

**S1.5 HDAC activity**

Cells were lysed and sonicated for 4 min and centrifuged at 13.000 rpm for 15 min at 4°C. 50 μg of whole lysates from each sample were used for determining HDAC activity. The assay has been performed following manufacturer’s instructions(BioVision).

**S1.6 Growth curves**

Cells grown either in GM or EpiC were detached with trypsin (Lonza) and counted in a Bürker chamber at days 3, 5, and 7 with Trypan Blue to verify their vitality.

**S1.7 Immunofluorescence**

Human cardiac mesenchymal-like stromal cells (CStC) (1 × 10^5^) were plated on 4-well chamber slide in presence of growth medium (GM) and Epigenetic Cocktail (EpiC) for 7 days. Cells were then fixed with 4% Paraformaldeide for 10 min. A blocking buffer (BSA at 5% in PBS1×) was added for 1 hr at room temperature. Primary antibodies for GATA4, c-Kit , and MDR-1 (see Supplementary Table 1) diluted in blocking buffer were added over night at 4°C in a humid chamber. A goat anti-rabbit secondary antibody labelled with Alexa 488 (1:200) was added for 1 hr at room temperature and nuclei were counterstained by DAPI. Slides were analyzed using an Axio Observer Z1 microscope, equipped with Apotome deconvolution system and software Axiovision (Zeiss).

**S1.8 Rhodamine 123 extrusion assay**

Efflux of Rhodamine 123 (Rh123) was analyzed as previously described by Neyfakh and co-workers [2]. Briefly, 500 μl of a suspension of 10^6^ cells/ml were incubated with 200 mg/ml of Rh123, in the presence or absence of Verapamil (Sigma Aldrich), for 45 min at 37°C. After centrifugation, the cells were washed in ice cold PBS and incubated again for 45 min at 37°C with CM and EpiC medium. Cell analysis was then performed on a FACSCalibur flow cytometer.

**Supplementary References**

1. Rossini A, Frati C, Lagrasta C, Graiani G, Scopece A, et al. (2011) Human cardiac and bone marrow stromal cells exhibit distinctive properties related to their origin. Cardiovasc Res 89: 650-660.

2. Neyfakh AA, Bidnenko VE, Chen LB (1991) Efflux-mediated multidrug resistance in Bacillus subtilis: similarities and dissimilarities with the mammalian system. Proc Natl Acad Sci U S A 88: 4781-4785.
